# Rationalising Exciton Interactions in Aggregates Based on the Transition Density

**DOI:** 10.1002/chem.202501570

**Published:** 2025-08-14

**Authors:** Joshua Krieger, Felix Plasser

**Affiliations:** ^1^ University of Münster Institute of Physical Chemistry Corrensstraße 28/30 48149 Münster Germany; ^2^ Department of Chemistry Loughborough University Loughborough LE11 3TU United Kingdom

**Keywords:** aggregates, excited states, exciton coupling, quantum chemistry, transition density

## Abstract

Aggregation effects of molecular chromophores play a crucial role in determining the spectroscopic properties of solid‐state organic materials. Within this work, we focus on excitonic coupling and particularly the question of whether aggregation leads to H‐ or J‐type coupling, that is, whether the lowest energy excited state of the aggregate is optically bright or not. Employing a supermolecular picture to represent the different terms giving rise to exciton splitting, we develop an intuitive and generally applicable phenomenological model for estimating the sign and magnitude of the exciton coupling. This model, which is based on the shape of the monomer transition density is shown to be suitable across the whole range of relevant wave function types from purely Coulomb‐coupled Frenkel excitons to strongly charge‐transfer admixed excimer states. The implications are illustrated in the stacked anthracene and perylene‐diimide dimer systems. The presented model does not only explain the long‐range behavior but provides a clear explanation of atom‐scale oscillations in the couplings seen for these systems. We hope that this work will give a boost to modern molecular materials science by providing new insight into interactions in the solid state as well as by highlighting the power of going beyond a simple frontier orbital picture in the design of molecular materials.

## Graphical abstract

Exciton coupling in organic chromophores is revisited through the lens of the transition density. The presented formalism gives insight into the strength and sign of the coupling based on the relative arrangement of the lobes of the transition density explaining oscillations between H‐ and J‐aggregate behavior observed when two molecules are displaced relative to each other.

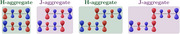

## 1 Introduction

Molecular materials play a crucial role in the quest for modern optoelectronic applications including light sources and solar cells.^[^ ^1^, ^2^, ^3^ ^]^ The devices used are essentially always in the solid state, meaning that aggregation effects can have a crucial influence. Not only does solid‐state packing act by confining and polarizing individual molecules but, importantly, interactions between different molecules can introduce crucial new physics via excitonic delocalization and trapping, charge transfer and charge separation.^[^ ^4^, ^5^, ^6^, ^7^ ^]^ Of particular interest in π‐stacked solids is the formation of H‐ and J‐aggregates resulting from different excitonic coupling interactions.^[^ ^3^, ^8^, ^9^, ^10^ ^]^ In the case of H‐aggregates the lowest state is dark whereas it is bright for J‐aggregates leading to markedly different spectroscopic signatures.^[^ ^11^, ^12^ ^]^ J‐aggregates, as discovered in the 1930s,^[^ ^13^, ^14^ ^]^ are distinguished by narrow red‐shifted absorption bands and strong fluorescence with a minimal Stokes shift.^[^ ^15^ ^]^ These properties make them favourable for OLEDs^[^ ^9^, ^16^ ^]^ and bioimaging applications.^[^ ^17^ ^]^ In addition, J‐aggregates have been implied for photovoltaics and photodetector applications.^[^ ^18^ ^]^ In light of the favourable properties of J‐aggregates, it is unfortunate that H‐aggregates are in many ways easier to construct considering that these are formed via simple sandwich‐type stacking. By contrast, the formation of J‐aggregates requires more specialised strategies, such as the fine‐tuning of non‐covalent interactions.^[^ ^15^, ^19^, ^20^ ^]^ Finally, fascinating new photophysics can be generated due to exciton coupling in covalently and mechanically bound supramolecular structures,^[^ ^21^, ^22^, ^23^ ^]^ further highlighting the importance of these phenomena.

Due to the importance of aggregation effects a range of computational methods have been developed to compute exciton couplings and these are applicable to describe optoelectronic materials as well as biological systems.^[^ ^24^, ^25^ ^]^ The starting point, following the initial ideas of Förster and Kasha,^[^ ^26^, ^27^ ^]^ is based on the interaction of the transition dipole moments. More sophisticated methods are based on electrostatic potential (ESP) fitting of the transition density, denoted TrESP charges,^[^ ^28^ ^]^ and the transition density cube method.^[^ ^29^ ^]^ Going beyond Coulomb interactions, exciton coupling models based on charge transfer integrals have been developed by Spano and co‐workers.^[^ ^8^, ^30^ ^]^ Finally, a number of authors have developed more sophisticated exciton coupling schemes making heavy use of available quantum chemistry tools.^[^ ^25^, ^31^, ^32^, ^33^, ^34^ ^]^

Whereas tremendous effort has, thus, been invested in obtaining the numerical values of exciton couplings, our phenomenological understanding of the underlying physics is not as advanced. The basic dipole‐dipole model explains why face‐to‐face stacks generally form H‐aggregates whereas head‐to‐tail arrangements yield J‐aggregates.^[^ ^20^ ^]^ However, it is much more difficult to rationalise atom‐scale oscillations in the exciton coupling, which have been reported for various systems.^[^ ^12^, ^30^, ^35^, ^36^ ^]^

The purpose of this work is to present a new framework for rationalising exciton coupling, going significantly beyond the dipole‐dipole model. To do so, we will employ a supermolecular picture, building on our previously developed framework for visualising excited states^[^ ^5^, ^37^ ^]^ and explaining their excitation energies.^[^ ^38^, ^39^, ^40^ ^]^ Specifically, we will base our analysis on the transition density, noting that the study of this quantity has a long‐standing tradition in quantum chemistry.^[^ ^41^, ^42^, ^43^ ^]^ While forming the basis for computing optical absorption strengths,^[^ ^37^, ^42^ ^]^ the transition density also has a relation to excited state energies contributing to excitonic coupling terms^[^ ^29^, ^42^ ^]^ as well as singlet‐triplet (*S*_1_/*T*_1_) energy gaps.^[^ ^38^, ^44^ ^]^ It, thus, provides an ideal starting point for studying the interplay between absorption strengths and excitation energies, which is at the heart of this work.

We first develop the mathematical formalism of our model in some detail explaining the origin of Coulomb and CT couplings within a supermolecular picture and highlighting how these terms can be understood intuitively. Subsequently, we present its implications for two crucial examples, the stacked anthracene and perylene‐diimide (PDI) dimers. In both cases, we displace the molecules from an eclipsed to a slip‐stacked arrangement studying how exciton coupling changes upon displacement. We highlight how an atomistic‐level view of the transition density explains the fine‐structure and oscillations in the couplings in a way that cannot be explained via a dipole model. We conclude this work by summarising the main design guidelines. The sections are largely self‐contained, so that readers not interested in the mathematical discussion may skip ahead directly.

## 2 Exciton Couplings Within a Supermolecule Picture

It is the purpose of this section to develop an intuitive phenomenological representation of exciton couplings between molecules. Here, we use the term “exciton” in its general sense describing any excitation in a dimer or aggregate. Below, we will discuss how much the observed excitons can be described as Frenkel excitons (that is, coupled local excitations) and when CT effects play a role. Here, we employ a top‐down supermolecule approach. We start our formal analysis based on time‐dependent density functional theory (TDDFT) computations performed on molecular dimers and decompose the excitation energies into various relevant energy terms. This provides an alternative approach to the more commonly employed bottom‐up approach based on monomer states interacting via perturbation theory.^[^ ^8^, ^28^, ^29^ ^]^ Where possible, we will draw connections between both viewpoints.

We will first discuss the mathematical framework building on our previous work regarding an energy decomposition for excitation energies.^[^ ^39^ ^]^ Next, we will provide a detailed discussion of the Coulomb coupling terms and develop practical rules, which are based on a graphical depiction of the transition densities. We will then discuss the influence of charge‐transfer (CT) contributions in some detail. In particular, we will elaborate on the connection between purely Coulomb‐coupled Frenkel excitons and strongly CT‐admixed excimer‐type states. From a practical point of view, this analysis will show that Coulomb and CT couplings can be approximated via the same phenomenological rules, providing one unified framework for all relevant terms.

### 2.1 Mathematical Framework

Our formal starting point is the TDDFT method, in which case the vertical excitation energy *E*_*x*_ of a given state can be written in the following way^[^ ^38^, ^39^ ^]^:

(1) $$\begin{array}{cc} E_{x} & =h^{\prime }+\underset{E_{2}}{\underset{︸}{J_{2}+K_{2}+XC_{2}}} \end{array}$$

(2) $$\begin{array}{cc} h^{\prime } & =\sum_{ia}{\left|C_{ia}\right|}^{2}\left(\varepsilon_{a}-\varepsilon_{i}\right) \end{array}$$

(3) $$\begin{array}{cc} J_{2} & =\;\int \int \;\frac{\rho_{t}\left(r_{1}\right)\rho_{t}\left(r_{2}\right)}{r_{12}}dr_{1}dr_{2} \end{array}$$

(4) $$\begin{array}{cc} K_{2} & =-c_{HF}\;\int \int \;\frac{|\gamma_{t}\left(r_{1},r_{2}\right){|}^{2}}{r_{12}}dr_{1}dr_{2} \end{array}$$

Here, *C*_*ia*_ are the CI coefficients and ϵ_*a*_ and ϵ_*i*_ are the energies of virtual and occupied orbitals, respectively. The function γ_*t*_ is the one‐electron ground‐to‐excited state transition density matrix (1TDM). In the case of TDDFT in the Tamm–Dancoff approximation (TDA), γ_*t*_ is constructed as

(5) $$\gamma_{t}\left(r_{h},r_{e}\right)=\sum_{ia}C_{ia}\phi_{i}\left(r_{h}\right)\phi_{a}\left(r_{e}\right)$$

where the functions ϕ_*i*_ (ϕ_*a*_) represent occupied (virtual) molecular orbitals (MOs). Here we use the coordinate labels *r*_*h*_ and *r*_*e*_ to emphasise that these can be identified with the excitation hole and excited electron in the quasiparticle exciton picture.^[^ ^45^ ^]^ The transition density ρ_*t*_ is given as the diagonal part of the 1TDM

(6) $$\rho_{t}\left(r\right)=\gamma_{t}\left(r,r\right)=\sum_{ia}C_{ia}\phi_{i}\left(r\right)\phi_{a}\left(r\right)$$

Furthermore, in the simplest case of a transition from the highest occupied MO (HOMO, ϕ_*H*_) to the lowest unoccupied MO (LUMO, ϕ_*L*_), the transition density is simply the product of these orbitals

(7) $$\rho_{t}\left(r\right)=\phi_{H}\left(r\right)\phi_{L}\left(r\right)$$

Going back to Equation (1), the first term *h*′ represents a weighted sum of MO energy differences. In the simplest case, this is just the HOMO/LUMO gap.

The remaining terms in Equation (1) are the post‐MO energy contributions. The first of these, *J*_2_, is computed as the self‐repulsion of the transition density. It is analogous to the ground‐state Hartree term (see, e.g., ref. [46]) only that the transition density is used instead of the density. This term is decisive for *S*_1_/*T*_1_ splittings,^[^ ^39^, ^44^ ^]^ and we will highlight its connection to the excitonic Coulomb coupling, below. *K*_2_ is the response of the non‐local exchange potential, present whenever a hybrid functional is used (with *c*_*HF*_ being the admixture of non‐local exchange). It is, again, computed in analogy to a corresponding ground state term.^[^ ^46^ ^]^ This term represents the Coulomb binding energy, relevant for excitonic and charge transfer states.^[^ ^39^, ^47^ ^]^ Finally, *XC*_2_ represents the response of the semilocal exchange‐correlation potential. The sum of the two‐electron terms will be denoted *E*_2_, as indicated in Equation (1).

Within the text to follow, we will also make use of the charge transfer number analysis^[^ ^5^, ^37^, ^48^ ^]^ to quantify CT mixing and charge resonance character. First the charge transfer number between two fragments *A* and *B* is defined as

(8) $$\Omega_{AB}=\int_{A}\int_{B}|\gamma_{t}\left(r_{h},r_{e}\right){|}^{2}dr_{e}dr_{h}$$

Using this, the overall amount of charge transfer of the excited state is computed by summing over all off‐diagonal CT numbers

(9) $$\text{CT}=\sum_{B\neq A}\Omega_{AB}$$

A non‐vanishing CT value can either refer to a simple CT state with a net transfer of charge from one chromophore to the other or to a charge resonance state^[^ ^5^, ^49^ ^]^ formed through simultaneous *A* → *B* and *B* → *A* transfers resulting in no net transfer of charge. Within the present work all dimers are symmetric, which means that per construction only symmetric charge resonance states are possible, and we will use the terms charge transfer and charge resonance interchangeably.

Finally, we note that the transition dipole moment, which forms the basis for computing absorption and emission strengths, is computed as the dipole moment of the transition density^[^ ^37^, ^42^ ^]^

(10) $$\vec{\mu_{t}}=\int \rho_{t}\left(r\right)\vec{x}dr$$

As alluded to in the Introduction, it is particularly noteworthy that the energy and transition dipole moment both depend on the transition density, as shown in Equations (3) and (10). We will use this interrelation to derive general rules for connecting energies and optical brightness to determine whether H‐ or J‐type couplings are present. We will first explain the emergence of Coulomb couplings via the *J*_2_ term and then investigate how the other terms give rise to CT couplings.

### 2.2 Coulomb Coupling

In the case of two interacting molecules (*A* and *B*), we rewrite the *J*_2_ term as

(11) $$J_{2}=J_{2}^{AA}+J_{2}^{BB}+2J_{2}^{AB}$$

where $J_{2}^{AB}$ marks the intermolecular contributions

(12) $$J_{2}^{AB}=\int_{A}\int_{B}\frac{\rho_{t}\left(r_{1}\right)\rho_{t}\left(r_{2}\right)}{r_{12}}dr_{1}dr_{2}$$

and the intramolecular contributions $J_{2}^{AA}$ and $J_{2}^{BB}$ are defined accordingly. In Equation (12) the integrals for coordinates *r*_1_ and *r*_2_ are restricted to fragments *A* and *B*, respectively. As initially pointed out in ref. [42], the $J_{2}^{AB}$ term gives rise to the Coulombic exciton coupling. Practically, one usually carries out the integration with transition densities obtained from isolated monomer calculations, for example, within the transition density cube method.^[^ ^29^ ^]^ Within the following, we will not evaluate it directly but use it only for interpreting the results of the supermolecule calculations performed. For ease of interpretation, we will discuss the case of a symmetric homodimer with evenly delocalised exciton states. In this case the Coulomb coupling can be defined as

(13) $$V_{AB}=2J_{2}^{AB}\text{(bright)}$$

Following the usual sign convention, a negative value for *V*_*AB*_ means that the bright state is lower than the dark state (i.e., J‐type coupling). This means that *V*_*AB*_ has the same sign as $J_{2}^{AB}$ determined for the bright state, as indicated in Equation (13). Furthermore, using the normalisation employed here, the Coulomb coupling is twice the $J_{2}^{AB}$ term.

Before going into a more detailed discussion, we point out that the basic approximation to the Coulomb coupling is given within a point‐dipole approximation^[^ ^27^ ^]^

(14) $$V_{AB}\approx \frac{{\vec{\mu }}_{t}^{A}\cdot {\vec{\mu }}_{t}^{B}-3\left({\vec{\mu }}_{t}^{A}\cdot {\vec{R}}_{0}\right)\left({\vec{\mu }}_{t}^{B}\cdot {\vec{R}}_{0}\right)}{R^{3}}$$

Here, ${\vec{\mu }}_{t}^{A}$ and ${\vec{\mu }}_{t}^{B}$ are the monomer transition dipole moments defined analogously to Equation (10), *R* is the distance between the monomers, and ${\vec{R}}_{0}$ is a unit vector pointing from the centre of one monomer to the centre of the other. The equation can be further simplified for the case of a homodimer and if the transition dipole moments are parallel to give^[^ ^27^, ^30^ ^]^

(15) $$V_{AB}\approx \frac{|{\vec{\mu }}_{t}{|}^{2}\left(1-3cos^{2}\theta \right)}{R^{3}}$$

where θ is the angle between the transition dipole moment vector and the line connecting the molecular centers. Equation (15) predicts J‐aggregate formation for θ < 54.7° and H‐aggregate formation for larger values^[^ ^27^ ^]^ and we will investigate this relation in more detail below.

The dipole–dipole approximation works well when the separation between the chromophores is significantly larger than their size, meaning that they can indeed be treated as point dipoles. However, it can fail for stacked aggregates where intermolecular separations are often smaller than the individual molecules.^[^ ^12^, ^30^ ^]^ Clearly a more fine‐grained model for understanding exciton coupling in such systems is needed.

**Figure 1:**
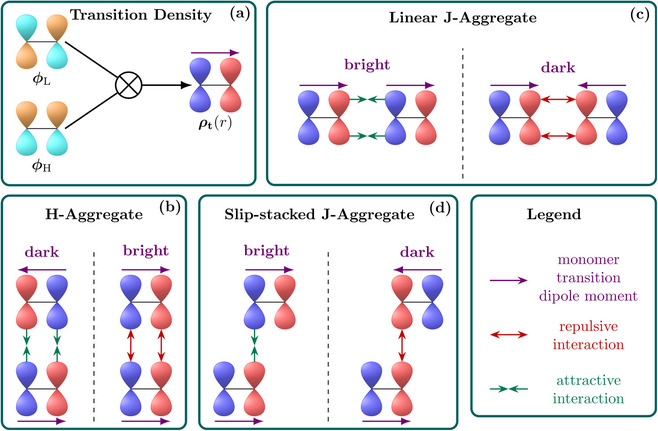
Phenomenological description of exciton coupling explained via transition density interactions: a) transition density formed as the direct product of the corresponding occupied and virtual orbitals; b) H‐aggregate present for face‐to‐face stacking; J‐aggregates for linear c) and slip‐stacked d) arrangements. The different color (cyan/orange for orbitals; red/blue for transition densities) represent the sign of the function.

Figure 1 lays out the framework for such a more fine‐grained model for rationalising exciton coupling between molecules using, here, still a purely Coulomb based picture. The different steps are represented pictorially using the toy model of an ethene molecule. Figure 1a illustrates that, for a simple orbital‐to‐orbital transition the transition density ρ_*t*_ can be formed as the product function of the *hole* and *electron* orbitals (typically the HOMO and LUMO) involved, see Equation (7). Following Equation (10), the transition dipole moment is the dipole moment of ρ_*t*_. Shown as a purple arrow in Figure 1a, it is constructed as the vector going from the center of the “blue” (negative) parts of ρ_*t*_ to the center of the “red” (positive) parts.

To explain the developed ideas, we will next proceed to various arrangements of the model of the two stacked molecules. The case of a fully eclipsed face‐to‐face sandwich stack is shown in Figure 1b with one ethene molecule at the top and the other at the bottom. We can form two possible combinations of the monomer transition densities. To the left in Figure 1b we show the antiparallel (red‐blue / blue‐red) arrangement; to the right we show the parallel (blue‐red / blue‐red) arrangement. First, it can be realised that the monomer transition dipole moments cancel out in the left case leading to a dark state whereas they interact constructively in the right case producing a bright state. We will now aim to determine which one of these states is higher in energy. Viewing the left case, we realise that the intermolecular *J*_2_‐interactions are predominantly attractive, since blue lobes on one molecule are near red lobes on the other. This attractive interaction is represented via green arrows. The opposite is true for the bright exciton shown to the right in Figure 1b. In this case the blue lobes and red lobes are in closer proximity to each other producing a predominantly repulsive interaction. Considering that the dark state has predominantly attractive interactions and the bright state predominantly repulsive interactions, it is clear that the bright state is higher in energy, i.e., the stack shown in Figure 1b forms an H‐aggregate.

The case of a linear arrangement is shown in Figure 1c. In this case similar considerations apply as just discussed for Figure 1b. However, the crucial difference is that the bright state now has predominantly attractive interactions whereas the dark state experiences repulsive interactions. The consequence of this is that the bright state is lower in energy and, hence, a J‐aggregate is formed.

Finally, a slip‐stacked arrangement is presented in Figure 1d. In the present example we choose the offset in such a way as to maximise the interaction of the right transition density lobe of the bottom molecule with the left transition density lobe of the top molecule. One can now appreciate that this arrangement will also form a J‐aggregate following the ideas discussed above, since the bright state experiences an attractive interaction and *vice versa* for the dark state. In the toy model of ethylene there are just two lobes per molecule and further displacement would just separate the molecules in space. For larger molecules one might anticipate oscillations in the couplings as lobes of different signs are moved past each other; this phenomenon will be investigated below.

We believe that Figure 1 provides a way of representing excitonic coupling interactions that is not only more detailed but also more intuitive than the standard dipole model. Nonetheless, it is also of interest how Figure 1 could be interpreted within the dipole approximation of Equation (15). The face‐to‐face stack of Figure 1b corresponds to an angle of θ = 90° and correctly leads to a positive *V*_*AB*_ term for the bright state indicating H‐type coupling. The head‐to‐tail arrangement of Figure 1c corresponds to an angle of θ = 0° associated to J‐type coupling. Within the dipole model, the transition between H‐ and J‐type coupling occurs at an angle of θ = 54.7° representing a slip‐stacked structure. The basic dipole model is, thus, able to provide a qualitative picture similar to Figure 1. However, below we will investigate oscillations in the couplings that are not reflected in the dipole model and indeed need atomistic information for their rationalisation.

The model developed above is based purely on Coulomb contributions, that is, the *J*_2_ term in Equation (1). Being inspired by the popular transition density cube^[^ ^29^ ^]^ and TrESP methods,^[^ ^28^ ^]^ we believe that it is certainly a powerful starting point. So far, the presented derivation does not explicitly include contributions from charge transfer or orbital overlap, and we will turn to these next.

### 2.3 Charge‐Transfer Contributions

**Figure 2:**
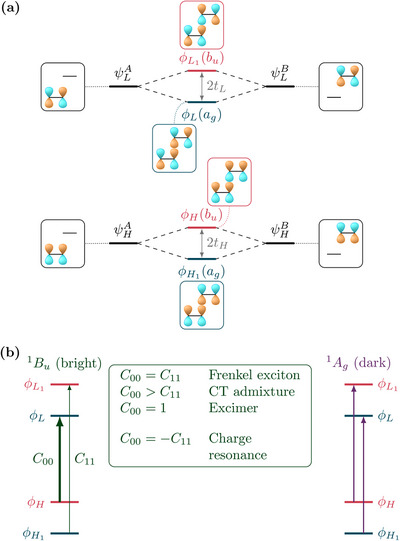
Origin of charge‐transfer contributions to the exciton splitting in a homodimer considering the case where the bright ^1^ *B*_*u*_ state becomes stabilised, thus, forming a J‐aggregate: a) splitting of the monomer frontier orbitals forming the dimer orbitals; b) orbital transitions leading to the bright and dark excitons.

The transition densities, as used in the previous section, provided a convenient means to summarise the relevant Coulomb interactions in a compact manner. Building up a phenomenological understanding beyond the Coulomb approximation is significantly more involved, as we need to keep track of the relative signs of the different orbitals involved. Figure 2 presents an attempt to do so. Due to the enhanced complexity, we only show the slip‐stacked case (corresponding to Figure 1d); the face‐to‐face stack is presented in Figure S1. We consider two symmetry‐equivalent molecules (*A* and *B*) in the dimer. For each molecule we consider the HOMO ($\psi_{H}^{A}$, $\psi_{H}^{B}$) and the LUMO ($\psi_{L}^{A}$, $\psi_{L}^{B}$). These orbitals are allowed to interact producing the dimer HOMO ($\phi_{H}$) and HOMO–1 ($\phi_{H_{1}}$) as well as the LUMO ($\phi_{L}$) and LUMO+1 ($\phi_{L_{1}}$). Within a symmetric dimer, these are constructed as (see also ref. [49])

(16) $$\begin{array}{cc} \phi_{H}\;/\;\phi_{H_{1}} & =\frac{1}{\sqrt{2}}\left(\psi_{H}^{A}\pm \psi_{H}^{B}\right) \end{array}$$

(17) $$\begin{array}{cc} \phi_{L}\;/\;\phi_{L_{1}} & =\frac{1}{\sqrt{2}}\left(\psi_{L}^{A}\pm \psi_{L}^{B}\right) \end{array}$$

The sign in the linear combination as well as the splitting between these MOs is determined by the hole (*t*_*H*_) and electron (*t*_*L*_) transfer integrals, defined as

(18) $$\begin{array}{c} t_{H}=\langle \psi_{H}^{A}|\hat{f}\left|\psi_{H}^{B}\right\rangle \end{array}$$

(19) $$\begin{array}{c} t_{L}=\langle \psi_{L}^{A}|\hat{f}\left|\psi_{L}^{B}\right\rangle \end{array}$$

where $\hat{f}$ is the Fock operator. The effect of *t*_*H*_ and *t*_*L*_ is indicated in Figure 2a and explained in more detail in Note S1.2.

The starting point for the qualitative argument made here is that, generally, the dimer MOs will transform as different irreducible representations (irreps) of the symmetry group of the dimer. For the sake of argument, we will assume that the dimer possesses an inversion center and that the relevant orbitals are either of *a*_*g*_ or *b*_*u*_ symmetry. The crucial consideration of whether the splitting of the MOs leads to J‐ or H‐type coupling now lies in the question of whether the transition from the dimer HOMO (ϕ_*H*_) to the dimer LUMO (ϕ_*L*_) is symmetry allowed or not. In the example of Figure 2a ϕ_*H*_ and ϕ_*L*_ are of *b*_*u*_ and *a*_*g*_ symmetry, respectively, meaning that the transition between them is allowed by the Laporte rule. Viewed from the opposite perspective, this now means that the bright state benefits from the reduced HOMO/LUMO gap and is lowered in energy. The opposite case, where the HOMO/LUMO transition leads to a dark state is presented in Figure S1. Note that the presented discussion uses arguments from symmetry group theory as this allows to discuss the underlying physics in a relatively compact way, following ideas originally developed by Spano and co‐workers.^[^ ^8^ ^]^ However, it should be pointed out that the underlying interaction terms do not rely on this symmetry and similar arguments can also be made without invoking symmetry.

Figure 2b shows the effect of the reduced HOMO/LUMO gap on the two excitonic states. Initially, there are four quasi‐degenerate transitions starting from $\phi_{H_{1}}$ and ϕ_*H*_ and going into ϕ_*L*_ and $\phi_{L_{1}}$. If the MO energy levels are not split, then the ^1^ *B*_*u*_ state is an even mixture between the ϕ_*H*_ → ϕ_*L*_ and $\phi_{H_{1}}\;\to \;\phi_{L_{1}}$ transitions while the ^1^ *A*_*g*_ state is an even mixture between the $\phi_{H_{1}}\;\to \;\phi_{L}$ and $\phi_{H}\;\to \;\phi_{L_{1}}$ transitions. In the present example, the lowering of the HOMO/LUMO gap means that the ^1^ *B*_*u*_ state obtains an enhanced contribution from the ϕ_*H*_ → ϕ_*L*_ transition. Conversely, the wave function (and energy) of the dark state remain largely unaffected, since the $\phi_{H_{1}}\;\to \;\phi_{L}$ and $\phi_{H}\;\to \;\phi_{L_{1}}$ transitions remain quasi‐degenerate.

We note that changes in MO energies generally stabilise one of the two states leaving the other one largely unaffected. In the case of *t*_*H*_ = *t*_*L*_, as indicated in Figure 2, only the bright state is stabilised leading to J‐type coupling; in the case of *t*_*H*_ = −*t*_*L*_, as indicated in Figure S1, only the dark state is stabilised leading to H‐type coupling. More generally, we can derive the following relation between the signs of the transfer integrals and the type of coupling (see Note S1.2).

(20) $$\begin{array}{c} \text{J-type coupling:}\;t_{H}\times t_{L}>0 \end{array}$$

(21) $$\begin{array}{c} \text{H-type coupling:}\;t_{H}\times t_{L}<0 \end{array}$$

These rules nicely align with the ones derived by Spano and co‐workers, who arrived at analogous conclusions using a perturbation theory approach starting from monomer states.^[^ ^8^ ^]^

In a next step, we were interested in how the above discussion relates to charge transfer. For this purpose, we first construct the 1TDM of the bright state

(22) $$\gamma_{t}\left(r_{1},r_{2}\right)=C_{00}\phi_{H}\left(r_{1}\right)\phi_{L}\left(r_{2}\right)+C_{11}\phi_{H_{1}}\left(r_{1}\right)\phi_{L_{1}}\left(r_{2}\right)$$

where *C*_00_ and *C*_11_ are the coefficients of the ϕ_*H*_ → ϕ_*L*_ and $\phi_{H_{1}}\;\to \;\phi_{L_{1}}$ transitions. After inserting Equations (16) and (17), we can express the 1TDM in terms of monomer orbitals

(23) $$\begin{array}{c} \gamma_{t}\left(r_{h},r_{e}\right)=\\ \frac{1}{2}\left(C_{00}+C_{11}\right)\underset{\text{LE contributions}}{\underset{︸}{\left[\psi_{H}^{A}\left(r_{h}\right)\psi_{L}^{A}\left(r_{e}\right)+\psi_{H}^{B}\left(r_{h}\right)\psi_{L}^{B}\left(r_{e}\right)\right]}}+\\ \frac{1}{2}\left(C_{00}-C_{11}\right)\underset{\text{CT contributions}}{\underset{︸}{\left[-\psi_{H}^{A}\left(r_{h}\right)\psi_{L}^{B}\left(r_{e}\right)+\psi_{H}^{B}\left(r_{h}\right)\psi_{L}^{A}\left(r_{e}\right)\right]}} \end{array}$$

The crucial realisation here is that this expression generally contains not only locally excited (LE) contributions but also CT contributions. As can be seen from Equation (23), the CT contributions only vanish if the HOMO/LUMO and HOMO–1/LUMO+1 transitions contribute with equal weight (that is, *C*_00_ = *C*_11_). This marks the limiting case of an idealised Frenkel exciton.^[^ ^5^, ^40^, ^49^, ^50^ ^]^ If the contribution of the HOMO/LUMO transition is enhanced (*C*_00_ > *C*_11_), then it follows that there is CT admixture. Furthermore, a pure HOMO/LUMO transition (*C*_00_ = 1) between delocalised orbitals implies that the state is a 50/50 mixture between LE and CT character. Such delocalised states with significant CT admixture have previously been identified as excimer states.^[^ ^5^, ^51^, ^52^, ^53^ ^]^ Finally, we note the potential limiting case of *C*_00_ = −*C*_11_. In this case, the LE contributions vanish and one obtains a pure charge resonance state (cf. ref. [5]). The four limiting cases are indicated in Figure 2b.

The above discussion highlights the importance of interference effects deriving from interacting electronic configurations, leading to correlation and entanglement effects. The interested reader is referred to ref. [54] for more details on this aspect.

Next, we were interested in determining how CT coupling is reflected within the excitation energy decomposition from Equation (1). Starting with the MO energy contributions, according to Equation (2), we find

(24) $$\begin{array}{cc} h_{\text{bright}}^{\prime } & =C_{00}^{2}\left(\varepsilon_{L}-\varepsilon_{H}\right)+C_{11}^{2}\left(\varepsilon_{L_{1}}-\varepsilon_{H_{1}}\right) \end{array}$$

(25) $$\begin{array}{cc} h_{\text{dark}}^{\prime } & =\frac{1}{2}\left(\varepsilon_{L_{1}}-\varepsilon_{H}\right)+\frac{1}{2}\left(\varepsilon_{L}-\varepsilon_{H_{1}}\right) \end{array}$$

for the bright and dark states. The difference between them can be expressed with the help of the charge transfer integrals as

(26) $$\Delta h^{\prime }=h_{\text{bright}}^{\prime }-h_{\text{dark}}^{\prime }=-\left(C_{00}^{2}-C_{11}^{2}\right)\left(|t_{H}\left|+\right|t_{L}|\right)$$

Here and in the following analysis, the Δ symbol is generally used to describe the difference between the bright and dark excited states, that is

(27) $$\Delta X=X_{\text{bright}}-X_{\text{dark}}$$

for any quantity *X* as defined in Equations (1)–(4).

Equation (26) highlights that, within our energy decomposition analysis, we expect CT‐induced exciton splitting to be primarily reflected in the *h*′ term.

A discussion of the two‐electron terms is more involved (see Note S1.1). We end up with the equation

(28) $$\begin{array}{c} \Delta E_{2}=\Delta J_{2}+\Delta K_{2}+\Delta XC_{2}=\\ \frac{1}{2}{\left(C_{00}-C_{11}\right)}^{2}\left[E_{\text{CT}}-E_{\text{LE}}-2{\tilde{J}}_{2}^{AB}\right] \end{array}$$

describing the combined effect of the two‐electron terms *J*_2_, *K*_2_, and *XC*_2_. In Equation (28) *E*_LE_ is the energy of the locally excited state on one monomer, and *E*_CT_ is the energy for a CT state going from one monomer to the other.

Note that *E*_LE_ and *E*_CT_ also appear in the formalism of Spano and co‐workers.^[^ ^8^ ^]^ The term ${\tilde{J}}_{2}^{AB}$ is the intermolecular Coulomb term that would have been obtained for the pure exciton states. The Δ*E*_2_ term, thus, involves an energetic penalty for the formation of partial CT character as well as a reduction in the Coulomb coupling because only the locally excited part of the wave function undergoes Coulomb coupling. The overall Δ*E*_2_ is generally opposed to *h*′ and the overall excited state can be seen as a compromise between the two driving forces.

Finally, reviewing Figure 2a, we note that the presented energy ordering of the MOs can indeed be deduced based on formal arguments, not requiring computations. For constructing the MO scheme and the excitonic states, it is enough to realise that in‐phase linear combinations of MOs are stabilised compared to out‐of‐phase linear combinations and to apply appropriate symmetry selection rules. Nonetheless, the scheme, as presented in Figure 2 does not seem to be by itself convenient for more complicated systems. The challenge for practical applications is that one needs to keep track of the relative phases and symmetry relations of four individual functions (the monomer MOs). It is, therefore, interesting to investigate whether the simpler transition density scheme of Figure 1 can also be used to estimate the sign of the CT couplings.

Indeed, we note that the slip‐stacked arrangement of Figure 2 yields J‐type coupling consistently with Figure 1d. Similarly, an eclipsed stacking arrangement, as presented in Figure S1, yields H‐type coupling. More generally, we outline in Note S1.3 and discuss in practical computations below that the sign and magnitude of the CT couplings can be approximated via the transition densities using the same relations derived for the Coulomb terms. This derives from the fact that the transition densities are computed as products of the underlying orbitals and Figure 2 can, thus, be compressed to the transition density picture. Consequently, we can use the rules developed above for all types of splittings. However, one should also keep in mind the differences. Coulomb couplings only apply to singlet states. They act evenly on the dark and bright exciton states, pushing one up and the other down. CT interactions affect singlet and triplet states alike. They are generally lowering the energies of excitonic states (while pushing up the higher energy CT states). CT couplings are expected to dominate for either the dark or the bright state, possibly leaving the other completely unaffected.

## 3 Computational Details

Structure optimisations of the anthracene and PDI monomers were performed using density functional theory (DFT) at the M06‐2X/def2‐TZVP level of theory.^[^ ^55^, ^56^ ^]^ The dimer stacks for these molecules were constructed by arranging the ground‐state optimized monomers according to the displacements specified. Vertical excitation energies were computed using time‐dependent DFT (TDDFT) in the Tamm–Dancoff approximation at the M06‐2X/def2‐SVP level,^[^ ^57^ ^]^ and these computations were also used for plotting transition densities. We always performed supermolecular computations on symmetric homodimers and identified the exciton splitting with the *S*_2_/*S*_1_ gap. We note that some of the presented results go significantly beyond the Coulomb coupling regime and some care is required when analysing these splittings. Nonetheless, the presented analysis is certainly adequate for the task of specifying whether the lowest state is bright or dark, which is the main focus of this work. Energy component analysis in line with Equation (1) was performed following refs. [39, 58]. All computations were performed using the q‐chem 6.1 program package^[^ ^59^ ^]^ using the libwfa library for transition density plots and further analysis.^[^ ^60^ ^]^

## 4 Results and Discussion

Having developed our formalism, we will now proceed to an analysis of two paradigmatic organic chromophores, anthracene and PDI and study their π‐stacked dimers in detail with respect to formation of H‐ and J‐aggregates.

### 4.1 Anthracene Dimer

**Figure 3:**
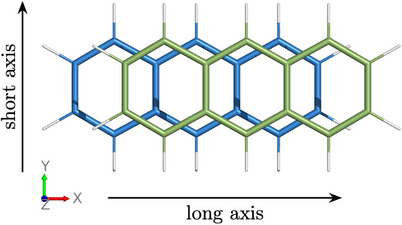
Depiction of the slip‐stacked anthracene dimer with an offset of *d*_*x*_ = 1.5 Å along the long molecular axis and definition of the coordinate system: the long axis is aligned with the x‐axis, the short axis with the y‐axis, the molecules are stacked along the z‐axis.

We start the discussion with a dimer of two anthracene molecules in a parallel stacking arrangement, investigating the eclipsed face‐to‐face stack as well as slip‐stacked arrangements obtained after displacing along either the short or long molecular axes. The coordinate system is illustrated in Figure 3. The first anthracene molecule was placed in the xy‐plane with the long and short molecular axes oriented along the x and y axes of the coordinate system, respectively. A second anthracene molecule was placed with 4 Å offset in the z‐direction, initially creating a perfectly symmetric face‐to‐face stack. This fully eclipsed face‐to‐face stack forms an H‐aggregate with an exciton splitting of 0.426 eV between the *S*_1_ and *S*_2_ states (see Table S1). This splitting is manifested by the lowering of the *S*_1_ vertical excitation energy, indicating the presence of a strongly bound excimer state, a phenomenon discussed in detail elsewhere for anthracene and related polycyclic hydrocarbons.^[^ ^5^, ^61^, ^62^, ^63^ ^]^ In regard to the underlying wave functions, we find that the *S*_1_ state is strongly dominated by the HOMO/LUMO transition (*C*_00_ = 0.98), consistent with an excimer‐type wave function (see Figure 2b).

**Figure 4:**
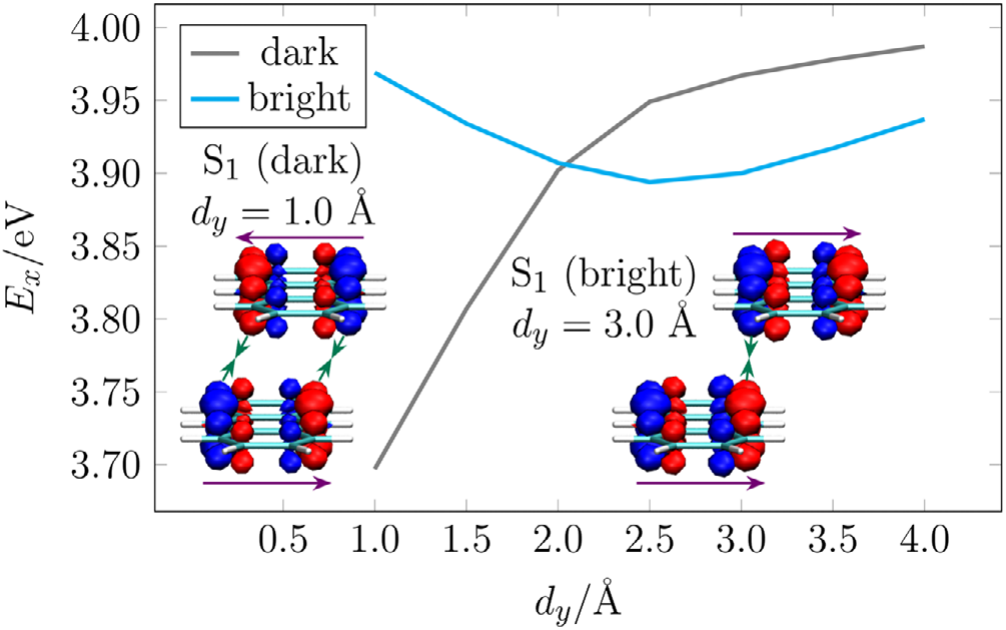
Analysis of the stacked anthracene dimer (*d*_z_ = 4.0 Å) with displacement along the short molecular axis: vertical excitation energies (*E*_*x*_) of the lowest dark and bright excited states; *S*_1_ transition density plots at 1.0 and 3.0 Å with monomer transition dipole moments as purple arrows.

We next investigate the effect of shifting one molecule along the short molecular axis (y‐axis), that is, in the direction of the monomer transition dipole moment, and the results are presented in Figure 4. In this figure, the dark state energy is shown as a grey line, the bright state in blue, and two exemplary *S*_1_ transition densities are shown as inset. Viewing the energies, we note a drastic decrease in the exciton splitting until the dark and bright states become degenerate at a shift of about *d*_*y*_ = 2.0 Å. After this crossing, the *S*_1_ becomes the bright state and a J‐aggregate is formed. The J‐type exciton splitting reaches a maximum of 0.067 eV at *d*_*y*_ = 3.0 Å after which the *S*_1_ and *S*_2_ energies both slowly converge toward the monomer excitation energy of 3.983 eV, ultimately expected to follow *R* ^−3^ asymptotics as indicated in Equation (14).

Having discussed the energies, we now proceed to a discussion of the transition density interactions. We first note that the fully ecplised face‐to‐face stack is analogous to the situation shown in Figure 1b (and Figure S1) with all lobes of the top and bottom molecules perfectly aligned. It is, thus, energetically favourable if the top transition density has its sign reversed with respect to the bottom one, which then leads to opposite directions of the transition moments and a dark *S*_1_ state. Within Figure 4, as inset shown to the left, we present the transition density of the dark *S*_1_ state at an offset of *d*_*y*_ = 1.0 Å. In this case, the left edge of the bottom molecule is still in close contact with the left edge of the top molecule (and similarly for the right edges) meaning that the dark state is energetically favourable similarly to the fully eclipsed stack; the dominant attractive interactions for the dark state are indicated via green arrows. The figure shown also indicates some minor repulsive (red‐red) interaction, but it should be noted that the corresponding lobes are displaced differently along the x‐axis meaning that they can only show minimal interaction here. The transition density of the bright *S*_1_ state at an offset of *d*_*y*_ = 3.0 Å is shown to the right. The dominant interaction is between the right edge of one anthracene molecule and the left edge of the other. In the *S*_1_ state both transition densities are oriented in the same way (blue to the left, red to the right), thus, producing constructive interference and a bright state. Thus, J‐type coupling is present in line with Figure 1d (and Figure 2).

Reviewing Figure 4, we note that our model of Figure 1 provides a clear explanation of the observed behavior. Next, it is also interesting to investigate whether the same qualitative behavior would be expected from the dipole approximation. Indeed, applying Equation (15) yields similar qualitative behavior with a swap from H‐ to J‐type coupling as the molecule is displaced. However, in the simple dipole model this swap would only occur at θ = 54.7° corresponding to a displacement of 2.8 Å whereas it occurs at 2.0 Å in the full computations. Moreover, the maximum J‐type coupling would occur at 4.0 Å in the dipole model, but it occurs at 3.0 Å in the full calculation. This latter point is marked by a maximal transition density interaction highlighting the importance of specific short‐range interactions over long‐range electrostatics.

**Figure 5:**
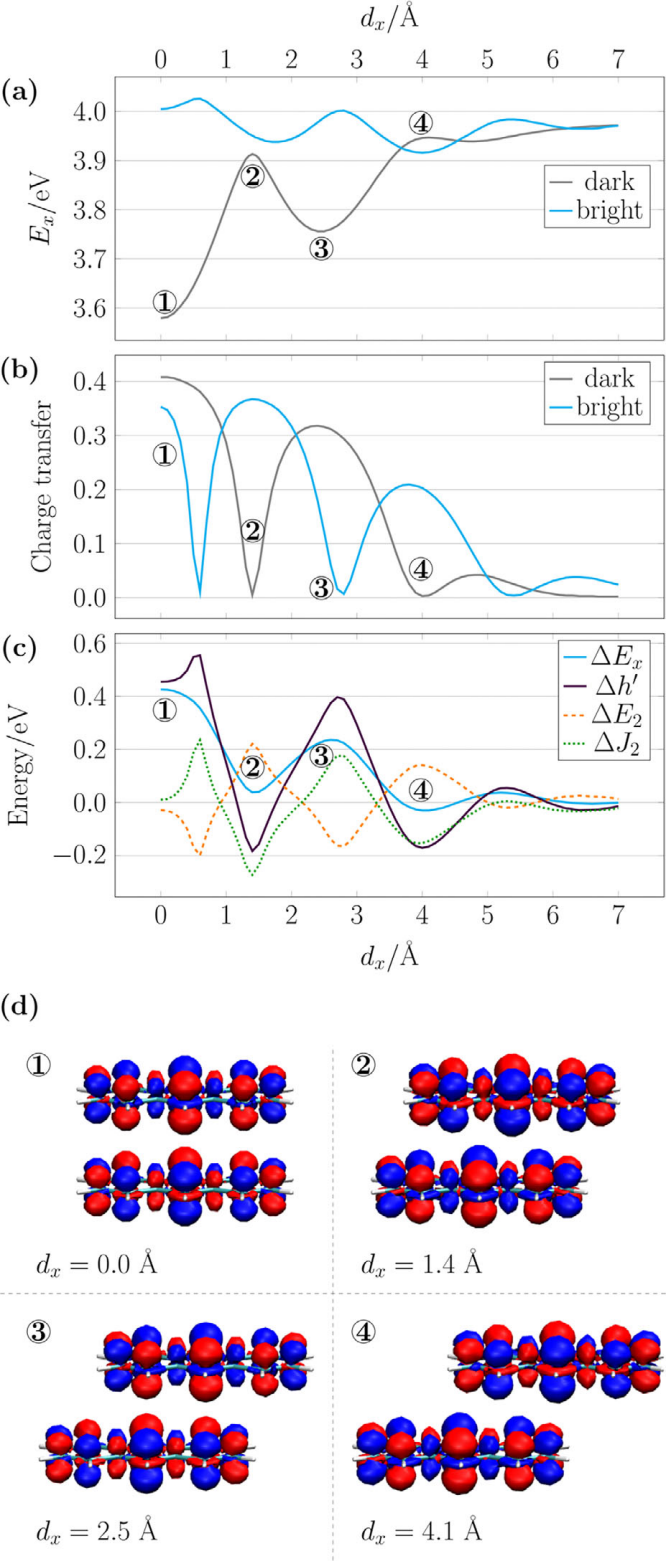
Analysis of the stacked anthracene dimer (*d*_z_ = 4.0 Å) with displacement along the long molecular axis: a) vertical excitation energies (*E*_*x*_) of the lowest dark and bright excited states; b) charge transfer character of these states; c) energy decomposition of the difference in excitation energy (Δ*E*_*x*_) into one‐electron (Δ*h*′), two‐electron (Δ*E*_2_) and pure Coulomb (Δ*J*_2_) terms; d) dark‐state transition density plots at 0.0, 1.4, 2.5, and 4.1 Å.

We, next, proceed to a somewhat more intricate case, obtained after displacement along the x‐axis (the long molecular axis), showing the energies in Figure 5a. The starting point is the same as before, the face‐to‐face stack with strong H‐type coupling, which is shown to the left side. Starting with the simple dipole model of Equation (15), we note that θ is equal to 90° throughout, meaning that the dipole model predicts a smooth *R* ^−3^ decay of the energy gap with the dark state always above the bright state. But, as Figure 5a shows, this is clearly not the case. Displacement along the x‐axis yields oscillatory behavior with a maximum of the dark state energy observed at 1.4 Å, then another minimum at 2.5 Å and finally a maximum at 4.1 Å. At larger displacements another oscillation is observed until the states almost converge after 6 Å and the exciton coupling vanishes. For most of these oscillations the system here is still an H‐aggregate and only in the area around 4.1 Å is there a brief area of J‐type behavior.

To explain the oscillations in the energies, we plot the transition densities at the extrema (labeled 1–4) considering always the dark state for consistency (Figure 5d). Crucially, we find that the extrema are always associated with the displacement of the top molecule by one atomic position with respect to the bottom one. One obtains maximum H‐type coupling for the perfect stack at 1 where the transition densities of the monomers align in a way such that lobes of opposite sign are next to each other, strongly stabilizing the dark state. The H‐type coupling is minimized at 2 where the top molecule is displaced such that the top blue lobes are now in closer proximity to the bottom blue lobes and similarly for the red lobes, thus, destabilizing the dark state. Enhanced H‐type coupling is again observed at 3 where red and blue lobes on the two molecules are now more clearly aligned. Finally, with the next displacement, yielding structure 4, J‐type coupling is obtained.

Viewing the transition densities in Figure 5d, we finally point out that, as expected these are predominantly focused on the π‐system. However, we note that there are also small contributions on the σ‐system, giving the overall atomic contributions the shape of $d_{z^{2}}$ orbitals. The origin of these well be discussed below.

Next we were interested in whether the observed energetic splitting is primarily due to Coulomb or charge transfer interactions. For this purpose, we plot the CT metric [Equation (9)] for the two states, as shown in Figure 5b. Interestingly, at the fully eclipsed face‐to‐face geometry, both states possess significant CT admixture, as alluded to above. For the low‐energy dark state, which is strongly dominated by the HOMO/LUMO transition, the CT admixture amounts to 0.41. For the higher energy bright state, dominated by the HOMO–1/LUMO transition, the CT admixture amounts to 0.35. Thus, both states possess excimer‐type wave functions as defined in Figure 2. Upon displacement, the CT character of the bright state rapidly drops reaching a value of 0.0 at *d*_*x*_ = 0.6 Å. At this geometry, the state becomes an even mixture between the HOMO–1/LUMO and HOMO/LUMO+1 transitions, thus, representing the Frenkel exciton case. The analysis around Figure 2 suggests that this downward spike in LE/CT mixing occurs when *t*_*H*_ = −*t*_*L*_, see also ref. [8]. Moving further along the displacement, we find additional spikes in CT character, which align with the extremal points discussed above. In addition, we find that CT character generally decreases along the displacement and only minimal contributions are found after about *d*_*x*_ = 5.0 Å. We note that the trends reported here reflect the findings from ref. [36].

Finally, we were interested in explaining the energy splitting via the excitation energy decomposition according to Equation (1), as shown in Figure 5c. The gap between the dark and bright states (Δ*E*_*x*_) is shown in light blue; Δ*E*_*x*_ follows the oscillations discussed earlier. Next, we plot the difference (Δ*h*′) between the one‐electron terms for the dark and bright states. This term can be seen as a signature of CT‐mediated energy splitting following Equation (26). The prominent role of Δ*h*′ is immediately apparent in Figure 5c; it follows the same oscillations as Δ*E*_*x*_, clearly serving as the dominant factor in determining the energy gap. The magnitude of Δ*h*′ is even larger than the overall energy gap, as it is compensated via two‐electron terms (Δ*E*_2_, orange dashes) reflecting the enhanced charge transfer character, following Equation (28). Viewing the pure two‐electron Coulomb terms (Δ*J*_2_, green dots), which contribute to Δ*E*_2_ along with Δ*K*_2_ and Δ*XC*_2_, we find that these mirror the overall Δ*E*_2_ but with opposite sign. Following Note S1.1 and particularly Equation (S12), we suggest that these changes in *J*_2_ reflect the charge transfer character of the individual states, rather than through‐space Coulomb coupling. In summary, we thus find that the energy splitting in this system is determined by CT interactions with only a secondary role of through‐space Coulomb coupling. A figure analogous to Figure 5a‐c for displacement in y‐direction is shown in Figure S2 also highlighting the importance of CT.

The above discussion highlights the importance of atom‐scale interactions in determining the energy splitting in the anthracene dimer, showing oscillations that could not be explained with a multipole model. We find that the energy splitting is largely determined by CT interactions with only smaller influence from through‐space Coulomb coupling. Nonetheless, we could show that the presented phenomenological model of aligning transition density lobes of different signs served as an excellent route for rationalising the oscillations observed.

### 4.2 PDI Dimer

We choose PDI as a next example to investigate exciton splitting following a similar approach as above, investigating displaced slip‐stacked dimers. PDI and its derivatives comprise a class of molecules that is widely used for dyes and optoelectronic building blocks; they are particularly known for their ability to form strongly coupled molecular H‐ and J‐aggregates.^[^ ^12^, ^64^ ^]^ Their excited‐state interactions have been investigated using a variety of computational protocols.^[^ ^4^, ^35^, ^65^, ^66^ ^]^

**Figure 6:**
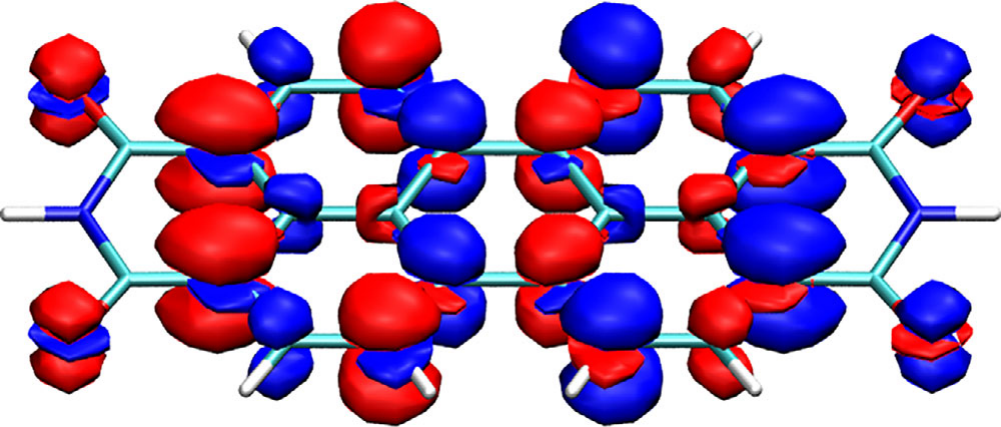
Structure of the perylene‐diimide (PDI) molecule along with the transition density of its *S*_1_ state.

The PDI molecule along with its transition density is shown in Figure 6. Viewing the transition density, it is noteworthy that the “red” parts are predominantly on the left and the “blue” parts on the right. In line with the rules developed, we can thus predict that PDI will have a strong transition dipole moment, going from right to left in Figure 6, aligned with the long molecular axis. Viewing Figure 6, we also find that the separation between blue and red parts is not perfect, i.e. there is blue interspersed in red and *vice versa*. As a consequence, we expect that the exciton coupling will depend on the precise alignment of the various lobes when the molecules are stacked.

Before proceeding, we want to discuss the precise shape of the transition density presented in Figure 6, focussing on the σ‐contributions already briefly mentioned in the context of Figure 5d. The *S*_1_ state of PDI is a ππ* state strongly dominated by its HOMO/LUMO transition. It is, thus, surprising to find contributions around the σ‐system. If the transition densities were formed as combinations of only π orbitals, then for every atom one would expect either only blue or only red contributions as indicated in Figure 1. But in fact, the individual atomic contributions resemble $d_{z^{2}}$ orbitals, containing a sign inversion around the σ‐system. We have previously highlighted that these additional contributions reflect the effect of σ correlation acting by lowering the *J*_2_ term (while raising *h*′) and reducing the transition dipole moments.^[^ ^38^ ^]^ These additional σ‐σ* excitations usually only represent a small contribution (<1%) to the overall excited state, but they are, nonetheless consistently found with various computational methods.^[^ ^38^, ^67^, ^68^ ^]^ Moreover, the absence of such σ‐contributions explains challenges for describing such ionic states with π‐only correlation models.^[^ ^67^, ^68^ ^]^ For the present qualitative discussion the presence of these σ‐contributions is not crucial but they should certainly be kept in mind for a quantitative treatment of all energy terms involved and highlight that it is challenging to rigorously separate between *J*_2_ and *h*′ type contributions.

In order to study exciton coupling we build a stacked dimer aligning the PDI molecules analogous to the anthracene molecules with the long axis being parallel to the x‐axis, the short axis parallel to the y‐axis and the molecules being stacked in z‐direction. We now choose a shorter stacking distance (*d*_*z*_ = 3.3 Å) consistent with the intermolecular separation obtained for the optimised dimer structure.

**Figure 7:**
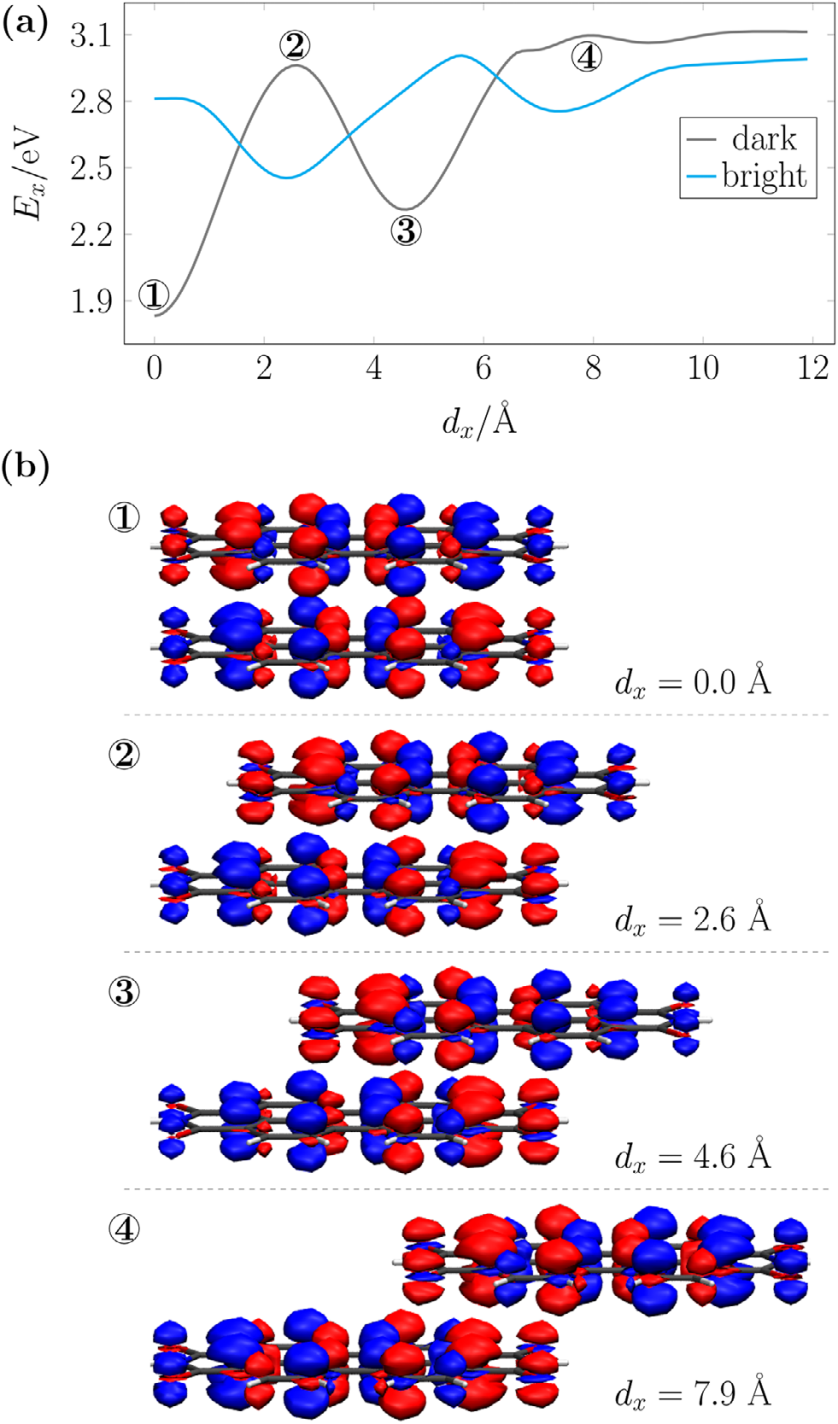
Analysis of the stacked PDI dimer (*d*_z_ = 3.3 Å) with displacement along the long molecular axis: a) energies of the lowest dark and bright excited states; b) dark‐state transition density plots at 0.0, 2.6, 4.6, and 7.9 Å.

Figure 7a presents the energies of the lowest dark and bright excited states of the PDI dimer as a function of the shift along the x‐axis, highlighting a similar profile to that observed for anthracene (Figure 5). Crucially, we obtain an oscillating profile exhibiting strong variations in the relative energies (see also refs. [12, 66]). Whereas the face‐to‐face stack exhibits strong H‐type coupling, the sign quickly reverses after the molecule is displaced and we find a J‐type maximum at *d*_*x*_ = 2.6 Å. Another H‐type extremum is found at 4.6 Å. Finally, after 6.2 Å  dominant J‐type coupling is observed and this remains when the molecules are further separated.

Transition density plots for the extremal points are shown in Figure 7b. We start with the face‐to‐face stacked dimer at position 1 where the dark state is stabilised by perfectly aligned transition density interactions and a maximum of H‐type coupling is obtained. Position 2, the J‐type maximum, represents a displacement by 2.6 Å, which is about the width of one benzene ring. Reviewing the monomer transition density in Figure 6, we notice that, if the transition density is moved to the right by the width of one benzene ring, then every red lobe lands on a blue lobe. As a consequence, the bright state is stabilised and J‐type coupling is obtained. The transition density for the dark state at position 2, as shown in Figure 7b, thus, highlights enhanced repulsive interactions. Note, however, that reading this figure is somewhat challenging due to the need to take into account the spatial depth in terms of the position of the different lobes. Position 3 is a displacement of 4.6 Å, which is about the width of one benzene ring plus an additional CC bond, meaning that the six‐membered rings are now, again, stacked on top of each other. The transition density plot highlights attractive red/blue interaction, stabilisation of the dark state and, thus, H‐type coupling. Position 4 represents the extended region of J‐type coupling at larger displacement. In the transition density plot, one finds that the extended red area of the bottom molecule aligns with the extended red area of the top molecule, thus also producing an extended area of J‐type coupling.

A more detailed analysis of CT contributions, in analogy to Figure 5b and c, is presented in Figure S3. For the dark state, we find a similar situation as before. It has about 50% CT character at the fully eclipsed geometry up until a displacement of about *d*_*x*_ = 6 Å, after which the CT character gradually decreases. The bright *S*_2_ state, interestingly starts out with pronounced charge resonance character (CT ≈1.0) and only becomes predominantly locally excited after *d*_*x*_ = 6 Å. This discussion highlights that the states can certainly not be completely described using a simple Coulomb model but, importantly, the qualitative discussion in terms of MO and transition density phases still applies.

**Figure 8:**
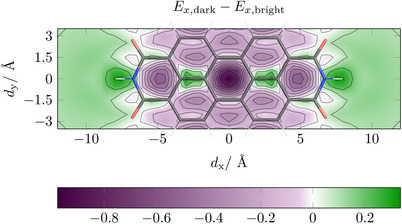
Energy difference in eV between the lowest dark and bright excited states of the PDI dimer (*d*_*z*_ = 3.3 Å). H‐type coupling is indicated in purple, J‐type coupling in green. The molecular structure overlaid indicates the position of the center of one PDI molecule relative to the other.

Finally, we were interested in also including a shift along the y‐axis into our analysis. For this purpose, we computed a two‐dimensional surface, simultaneously varying *d*_*x*_ and *d*_*y*_. A contour plot of the exciton splitting thus obtained is shown in Figure 8 with H‐type coupling in purple and J‐type coupling in green. The PDI molecule overlaid is used to indicate the relative position of the top molecule with respect to the center of the bottom one. In line with expectations we find that the strongest H‐type coupling is at the very center of the plot, which reflects the fully eclipsed face‐to‐face stack. Moving to the left and right on the *d*_*y*_ = 0 line reflects the data shown in Figure 7 noting oscillatory behavior and the alterations between H‐ and J‐type coupling. Moving up and down in Figure 8, the effect of short‐axis displacement is shown. Viewing the figure as a whole, we note an interesting pattern: there is an H‐type maximum near the center of every 6‐membered ring (along with the half‐ring at the armchair edge at top and bottom). We can explain this phenomenon by reviewing Figure 6. We note that every ring has the same sign of the transition density contributions (unless they vanish). When read from left to right the pattern is always: red / blue‐blue / red‐red / blue. Thus, if two rings are directly on top of each other, same sign contributions will be on top of each other, leading to a repulsive interaction for the bright state. Conversely, we note that regions of J‐type coupling or reduced H‐type coupling are generally shown along the bonds in this plot. Finally, we note a region of extended J‐type coupling on the left and right sides of this plot, representing a slip of more than half the molecular length. In these cases, there is not much variation in the behavior since the edges are predominantly blue and red, respectively.

As a practical consequence of Figure 8, we can conclude that if maximal H‐type coupling is intended, then, as expected, the fully eclipsed face‐to‐face stack is best. If J‐type coupling is required, then it is in principle possible to obtain this in the region of *d*_*x*_/*d*_*y*_ = 0.0/2.5 Å. But this has to be achieved with very high precision. More realistically, J‐aggregates can be achieved after displacement of about half the molecular length, leaving more tolerance for errors.

## 5 Outlook

**Figure 9:**
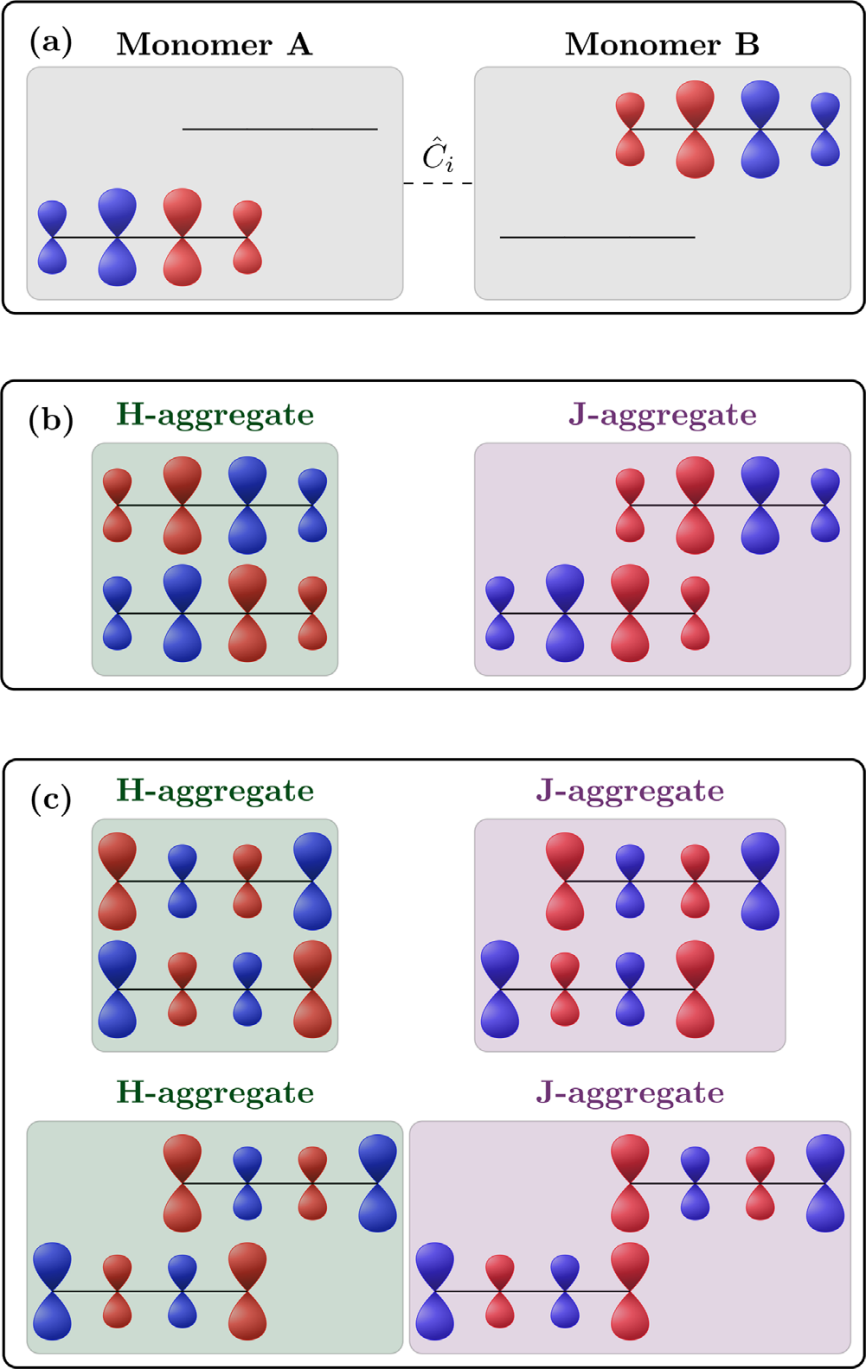
General guidelines for generating H‐ and J‐aggregates: a) sign convention for the monomer transition densities; b) case of a transition density with a simple dipolar shape; c) more complicated case with several nodes in the transition density.

Having presented the theory as well as specific concrete results in some detail, we now want to summarise the main findings and generate practical rules to be used in the design of H‐ and J‐aggregates. The main considerations are summarised in Figure 9. In the first step, as shown in Figure 9a (left), we need to construct the monomer transition density. This task can be easily achieved using most standard quantum chemistry codes. Alternatively, the transition density can be constructed using qualitative rules, thus, not requiring any computation at all.^[^ ^38^, ^41^, ^44^ ^]^ In any case, once the transition density is available, the remaining steps can be carried out not requiring a quantum chemistry computation. Once the transition density of monomer *A* is constructed, we need to project it onto monomer *B*. During this step we need to introduce an appropriate phase convention. Within the following, for the sake of argument, we will assume that the transition density of monomer *B* is created via an inversion or rotation operation, so that it is polarised in the opposite way as the one of monomer *A*, see Figure 9a (left). If the transition density on monomer *B* is generated via such a symmetry operation, then it follows automatically that its transition dipole moment is antiparallel to the one of *A*. The resulting exciton state is completely optically dark. We can now use the transition density interactions to tune the dark‐state energy (noting that the bright state behaves in exactly the opposite way). Alternatively, one can, of course, use the opposite construction and tune the bright state.

Once the transition densities are set up, we can proceed to the construction of various aggregates. The one general rule is: H‐aggregates are formed by maximising intermolecular red/blue and blue/red interactions. J‐aggregates are formed by maximising blue/blue and red/red interactions. We present two general cases, the one of a transition density with a simple dipolar shape (Figure 9b) as well as the case of a more complicated nodal structure (Figure 9c). In both cases, we note that an eclipsed arrangement maximises blue/red and red/blue interactions and, thus, produces strong H‐type coupling. In the first case, a J‐aggregate can be produced in one obvious way and that is by shifting the top molecule by half the length of the bottom molecule. This aligns the red lobes of both transition densities producing pronounced J‐type coupling. More generally, according to Figure 9b, one expects that displacement of the top molecule leads to a smooth transition from H‐type to J‐type behavior, as exemplified in Figure 4. The situation is more complicated in the case of Figure 9c where the transition density has more nodes. In this case, we can generate J‐type coupling by shifting the top molecule by one interatomic separation length. Shifting it by another interatomic separation yields again H‐type coupling, and finally J‐type coupling is reached if the outer atoms are aligned.

Figure 9 presents a one‐dimensional representation of aggregates. In practice, one of course has to include a second dimension for the molecule (along with a third dimension for the intermolecular separation). Nonetheless, the main rules remain the same; one needs to maximise the respective interactions of transition density lobes to achieve the desired type of coupling. It is noteworthy in this context, that strong H‐type coupling can always be achieved by simply generating a completely eclipsed arrangement where the matching lobes are always in close contact. As opposed to H‐type coupling, in two dimensions it may not be possible to achieve the idealised red/red with blue/blue alignment shown in the upper right of Figure 9c and, thus, strong J‐type coupling. J‐aggregates are clearly the more challenging case to design.

Whereas the previous discussion revolved around translations of the monomers, we also want to comment on rotation. The rules for the transition density lobes also apply for rotated molecules. However, after rotation, the transition dipole moments will no longer be aligned. As a consequence, it will not be possible to generate one completely dark and one bright exciton state.

Figure 9 can not only be read in the sense of predicting which type of intermolecular arrangement leads to which type of aggregate for a given molecule. It can also be read the other way: which type of molecule is best suited for inducing a given type of aggregate behavior? A molecule with pronounced dipolar shape of the transition density, as shown in Figure 9b, will have one extended region of enhanced H‐type coupling along with a region of enhanced J‐type coupling. If the goal is to maximise the H‐ or J‐type behavior of the aggregate in a robust fashion, then such molecules would be favourable. Conversely, a molecule with a more nodal structure of the transition density, as shown in Figure 9c, will have pronounced oscillations between H‐ and J‐type behavior. If the intermolecular arrangement can be tuned very finely, then this might allow for aggregates with maximal and tuned exciton coupling. Such molecules could also be expected to have a more intricate dynamical photophysical behavior where slight changes in the intermolecular arrangement can produce strong changes in the excited states. Conversely, such molecules are probably more challenging to use if the intention is to produce a more defined and robust aggregation behavior. Clearly, any given molecule does not have to conform with one limiting case completely. Taken anthracene as an example, it behaves more like the dipolar case when displaced along the short molecular axis (see Figure 4) and more like the oscillating case when displaced along the long molecular axis (see Figure 5). PDI behaves more like the oscillating case for small displacements changing to a smoother J‐type behavior after 6 Å offset.

In this context, we note that the differentiation between more dipolar and oscillating transition density patterns was also invoked by Chen and Cheng^[^ ^69^ ^]^ to explain trends in reorganisation energies. Combined with the power of the transition density to explain variations in singlet‐triplet gaps in conjugated hydrocarbons,^[^ ^44^ ^]^ and its ability to provide a lens through which to view excited state character,^[^ ^40^, ^41^ ^]^ we believe that the transition density is a highly suitable quantity for designing and understanding chromophore behavior providing important new insight on top of the traditional HOMO/LUMO picture.

Finally, we want to comment on the relevance of the presented discussion for larger aggregates, going beyond dimers. It is common practice to build exciton Hamiltonians for larger aggregates from pairwise nearest neighbour interactions.^[^ ^70^, ^71^ ^]^ This approach is also suitable for the presented formalism: individual dimer contributions can be estimated as described above and, to describe the aggregate, these have to be combined via an exciton Hamiltonian.

## 6 Conclusion

Within this work, a model was developed for rationalising exciton splitting in dimers based on the shape of the transition density. Using this model, the presence of H‐type and J‐type coupling can be explained in a quite intuitive fashion based solely on the relative positions of the transition density lobes on the interacting molecules. The model does not only explain the large‐scale behavior but provides a clear explanation of the otherwise puzzling presence of oscillations in the couplings depending on atom‐scale displacements of the molecules. While our model was initially derived based on an analysis of short‐range Coulomb coupling terms, we highlighted that the same overall relations also apply to CT couplings. In this context, we illustrated the transition from Frenkel excitons to emerging CT interactions to strongly CT‐admixed excimer‐type states, present for different intermolecular arrangements.

We started the discussion with the stacked anthracene dimer. It was first shown how a transition from H‐type to J‐type coupling occurs upon displacement along the short molecular axis. More specifically, we could show that the maxima of the respective H‐ and J‐type couplings coincided with the alignment of the relevant transition density lobes on the two molecules. Displacement along the long axis in the anthracene dimer showed intriguing oscillating behavior and we could rationalise this finding based on the alignment of different‐sign lobes of the transition density. A more detailed analysis of the involved wave functions showed the importance of CT admixture, especially when the molecules were strongly overlapping. Moving to the stacked perylene‐diimide (PDI) dimer, we found similar oscillations that could also be explained based on the shape of the monomer transition density and its alignment between the two molecules. Furthermore, a 2D‐plot showed how the relative alignment relates to the molecular structure. Maximum H‐type coupling was observed whenever the central 6‐membered ring of the top molecule was positioned just above a 6‐membered ring of the bottom molecule while J‐type couplings were found above some of the bonds. This behavior was rationalised based on the shape and associated signs of the monomer transition density.

After the practical examples, we presented general rules for how the insight gained could be translated into practical design guidelines. This analysis focused specifically on differentiating between molecules with a more dipolar vs a more oscillating shape of the transition density. Here, the former are more well‐behaved allowing to engineer the desired aggregates in a more robust fashion. Conversely, the latter might give rise to more extreme exciton splitting and more intricate photophysics but are probably more difficult to control. In the future, we hope this work will support researchers in the quest for engineering aggregates for molecular materials applications while also inspiring theoretical investigations into the intricate underlying wave functions.

## Conflict of Interest

No conflicts to declare.

## Supporting information

Supporting Information

## Data Availability

The data that support the findings of this study are openly available in Loughborough University's research repository at https://doi.org/10.17028/rd.lboro.28212647

## References

[1] T. M. Clarke , J. R. Durrant , Chem. Reviews 2010, 110, 6736. 10.1021/cr900271s 20063869

[2] H. Bronstein , Z. Chen , R. S. Ashraf , W. Zhang , J. Du , J. R. Durrant , P. S. Tuladhar , K. Song , S. E. Watkins , Y. Geerts , M. M. Wienk , R. A. Janssen , T. Anthopoulos , H. Sirringhaus , M. Heeney , I. McCulloch , J. the American Chem. Society 2011, 133, 3272. 10.1021/ja110619k 21332134

[3] O. Ostroverkhova , Chem. Rev. 2016, 116, 13279. 27723323 10.1021/acs.chemrev.6b00127

[4] R. F. Fink , J. Seibt , V. Engel , M. Renz , M. Kaupp , S. Lochbrunner , H. M. Zhao , J. Pfister , F. Würthner , B. Engels , J. Am. Chem. Soc. 2008, 130, 12858. 18767851 10.1021/ja804331b

[5] F. Plasser , H. Lischka , J. Chem. Theory Comput. 2012, 8, 2777. 26592119 10.1021/ct300307c

[6] M. Dommett , M. Rivera , R. Crespo‐Otero , J. Phys. Chem. Lett. 2017, 8, 6148. 29219318 10.1021/acs.jpclett.7b02893

[7] O. P. Dimitriev , Chem. Rev. 2022, 122, 8487. 35298145 10.1021/acs.chemrev.1c00648

[8] H. Yamagata , C. M. Pochas , F. C. Spano , J. Phys. Chem. B 2012, 116, 14494. 23194082 10.1021/jp309407r

[9] S. Ma , S. Du , G. Pan , S. Dai , B. Xu , W. Tian , Aggregate 2021, 2, e96.

[10] Q. Zhao , F. He , J. Energy Chem. 2024, 93, 174.

[11] F. C. Spano , Acc. Chem. Res. 2010, 43, 429. 20014774 10.1021/ar900233v

[12] M. Hecht , F. Würthner , Acc. Chem. Res. 2021, 54, 642. 33289387 10.1021/acs.accounts.0c00590

[13] E. E. Jelley , Nature 1936, 138, 1009.

[14] G. Scheibe , Kolloid‐Zeitschrift 1938, 82, 1.

[15] F. Würthner , T. E. Kaiser , C. R. Saha‐Möller , Angew. Chem. Int. Ed. 2011, 50, 3376. 10.1002/anie.201002307 21442690

[16] W. Li , W. Li , L. Gan , M. Li , N. Zheng , C. Ning , D. Chen , Y. C. Wu , S. J. Su , ACS Appl. Mater. Inter. 2020, 12, 2717. 10.1021/acsami.9b17585 31850735

[17] X. Hu , C. Zhu , F. Sun , Z. Chen , J. Zou , X. Chen , Z. Yang , Adv. Mater. 2024, 36, 2304848. 10.1002/adma.202304848 37526997

[18] J. H. Kim , T. Schembri , D. Bialas , M. Stolte , F. Würthner , Adv. Mater. 2022, 34, 2104678. 10.1002/adma.202104678 34668248

[19] M. E. Ziffer , S. B. Jo , Y. Liu , H. Zhong , J. C. Mohammed , J. S. Harrison , A. K. Jen , D. S. Ginger , J. Phys. Chem. C 2018, 122, 18860.

[20] Y. Tian , D. Yin , L. Yan , WIREs: Nanomed. Nanobiotech. 2023, 15, e1831. 10.1002/wnan.1831 35817462

[21] L. J. Patalag , L. P. Ho , P. G. Jones , D. B. Werz , J. Am. Chem. Soc. 2017, 139, 15104. 28948783 10.1021/jacs.7b08176

[22] L. Yang , P. Langer , E. S. Davies , M. Baldoni , K. Wickham , N. A. Besley , E. Besley , N. R. Champness , Chem. Sci. 2019, 10, 3723. 31015916 10.1039/c9sc00167k PMC6457202

[23] S. E. Penty , M. A. Zwijnenburg , G. R. F. Orton , P. Stachelek , R. Pal , Y. Xie , S. L. Griffin , T. A. Barendt , J. Am. Chem. Soc. 2022, 144, 12290. 35763425 10.1021/jacs.2c03531 PMC9348826

[24] G. D. Scholes , X. J. Jordanides , G. R. Fleming , J. Phys. Chem. B 2001, 105, 1640.

[25] C. Curutchet , B. Mennucci , Chem. Reviews 2017, 117, 294. 10.1021/acs.chemrev.5b00700 26958698

[26] T. Förster , Ann. Phys. 1948, 437, 55.

[27] M. Kasha , H. R. Rawls , M. A. El‐Bayoumi , Pure Appl. Chem. 1965, 11, 371.

[28] M. E. Madjet , A. Abdurahman , T. Renger , J. Phys. Chem. B 2006, 110, 17268. 16928026 10.1021/jp0615398

[29] B. P. Krueger , G. D. Scholes , G. R. Fleming , J. Phys. Chem. B 1998, 102, 5378.

[30] N. J. Hestand , F. C. Spano , J. Chem. Phys. 2015, 143, 244707. 26723702 10.1063/1.4938012

[31] C. P. Hsu , G. R. Fleming , M. Head‐Gordon , T. Head‐Gordon , J. Chem. Phys. 2001, 114, 3065.

[32] A. F. Morrison , J. M. Herbert , J. Phys. Chem. Lett. 2015, 6, 4390. 26538050 10.1021/acs.jpclett.5b02109

[33] A. Kaiser , R. E. Daoud , F. Aquilante , O. Kühn , L. D. Vico , S. I. Bokarev , J. Chem. Theory Comput. 2023, 19, 2918. 37115036 10.1021/acs.jctc.3c00185

[34] T. Pitesa , S. Polonius , L. González , S. Mai , J. Chem. Theory Comput. 2024, 20, 5609. 38885637 10.1021/acs.jctc.4c00157 PMC11238547

[35] W. Liu , S. Canola , A. Köhn , B. Engels , F. Negri , R. F. Fink , J. Comput. Chem. 2018, 39, 1979. 30315587 10.1002/jcc.25374

[36] Y. Dai , A. Calzolari , M. Zubiria‐Ulacia , D. Casanova , F. Negri , Molecules 2023, 28. 10.3390/molecules28010119 PMC9822017 36615311

[37] F. Plasser , M. Wormit , A. Dreuw , J. Chem. Phys. 2014, 141, 024106. 25027998 10.1063/1.4885819

[38] P. Kimber , F. Plasser , Phys. Chem. Chem. Phys. 2020, 22, 6058. 32154539 10.1039/d0cp00369g

[39] P. Kimber , F. Plasser , J. Chem. Theory Comput. 2023, 19, 2340. 37022304 10.1021/acs.jctc.3c00125 PMC10134415

[40] P. Kimber , F. Plasser , Classification and Analysis of Molecular Excited States, in L. González , H. Kulik (Editors), Comprehensive Computational Chemistry Vol. 4, Elsevier 2024, pp. 55.

[41] J. R. Platt , J. Chem. Phys. 1949, 17, 484.

[42] H. C. Longuet‐Higgins , Proc. R. Soc. A 1956, 235, 537.

[43] I. Fischer‐Hjalmars , J. Mol. Spectrosc. 1971, 39, 321.

[44] W. Zeng , C. Zhong , H. Bronstein , F. Plasser , Angew. Chem. Int. Ed. 2025, 64, e202502485. 10.1002/anie.202502485 PMC12087868 40062484

[45] S. A. Bäppler , F. Plasser , M. Wormit , A. Dreuw , Phys. Rev. A 2014, 90, 052521. 10.1063/1.4885820 25027999

[46] M. Casida , M. Huix‐Rotllant , Annu. Rev. Phys. Chem. 2012, 63, 287. 22242728 10.1146/annurev-physchem-032511-143803

[47] S. A. Mewes , F. Plasser , A. Dreuw , J. Phys. Chem. Lett. 2017, 8, 1205. 28230997 10.1021/acs.jpclett.7b00157

[48] A. V. Luzanov , O. A. Zhikol , Int. J. Quantum Chem. 2010, 110, 902.

[49] A. L. East , E. C. Lim , J. Chem. Phys. 2000, 113, 8981.

[50] I. Mayer , Chem. Phys. Lett. 2007, 443, 420.

[51] F. Plasser , H. Lischka , Photochem. Photobiol. Sci. 2013, 12, 1440. 23737069 10.1039/c3pp50032b

[52] V. A. Spata , W. Lee , S. Matsika , J. Phys. Chem. Letters 2016, 7, 976. 10.1021/acs.jpclett.5b02756 26911276

[53] Y. J. Bae , D. Shimizu , J. D. Schultz , G. Kang , J. Zhou , G. C. Schatz , A. Osuka , M. R. Wasielewski , J. Phys. Chem. A 2020, 124, 8478. 32975426 10.1021/acs.jpca.0c07646

[54] F. Plasser , J. Chem. Phys. 2016, 144, 194107. 27208936 10.1063/1.4949535

[55] Y. Zhao , D. G. Truhlar , Theor. Chem. Acc. 2008, 120, 215.

[56] F. Weigend , R. Ahlrichs , Phys. Chem. Chem. Phys. 2005, 7, 3297. 16240044 10.1039/b508541a

[57] S. Hirata , M. Head‐Gordon , Chem. Phys. Lett. 1999, 314, 291.

[58] Z. Pei , Q. Ou , Y. Mao , J. Yang , A. D. L. Lande , F. Plasser , W. Liang , Z. Shuai , Y. Shao , J. Phys. Chem. Letters 2021, 12, 2712. 10.1021/acs.jpclett.1c00094 PMC8272082 33705139

[59] E. Epifanovsky , A. T. B. Gilbert , X. Feng , J. Lee , Y. Mao , N. Mardirossian , P. Pokhilko , A. F. White , M. P. Coons , A. L. Dempwolff , Z. Gan , D. Hait , P. R. Horn , L. D. Jacobson , I. Kaliman , J. Kussmann , A. W. Lange , K. Un Lao , D. S. Levine , J. Liu , S. C. McKenzie , A. F. Morrison , K. D. Nanda , F. Plasser , D. R. Rehn , M. L. Vidal , Z. Q. You , Y. Zhu , B. Alam , B. J. Albrecht , A. Aldossary , et al., J. Chem. Phys. 2021, 155, 084801.34470363

[60] F. Plasser , A. I. Krylov , A. Dreuw , WIREs Comp Mol Sci 2022, 12, e1595.

[61] E. S. S. Iyer , A. Sadybekov , O. Lioubashevski , A. I. Krylov , S. Ruhman , J. Phys. Chem. A 2017, 121, 1962. 28182435 10.1021/acs.jpca.7b01070

[62] V. Vijay , M. Madhu , R. Ramakrishnan , A. Benny , M. Hariharan , Chem. Commun. 2020, 56, 225. 10.1039/c9cc07251a 31803867

[63] T. M. Cardozo , A. P. Galliez , I. Borges , F. Plasser , A. J. A. Aquino , M. Barbatti , H. Lischka , Phys. Chem. Chem. Phys. 2019, 21, 13916. 30570626 10.1039/c8cp06354k

[64] A. Oleson , T. Zhu , I. S. Dunn , D. Bialas , Y. Bai , W. Zhang , M. Dai , D. R. Reichman , R. Tempelaar , L. Huang , F. C. Spano , J. Phys. Chem. C 2019, 123, 20567.

[65] W. Liu , B. Lunkenheimer , V. Settels , B. Engels , R. F. Fink , A. Köhn , J. Chem. Phys. 2015, 143, 084106. 26328817 10.1063/1.4929352

[66] S. Canola , G. Bagnara , Y. Dai , G. Ricci , A. Calzolari , F. Negri , J. Chem. Phys. 2021, 154, 124101. 33810656 10.1063/5.0045913

[67] S. A. do Monte , R. F. Spada , R. L. Alves , L. Belcher , R. Shepard , H. Lischka , F. Plasser , J. Phys. Chem. A 2023, 127, 9842. 37851528 10.1021/acs.jpca.3c05559 PMC10683019

[68] J. C. Chagas , L. G. F. dos Santos , R. Nieman , A. J. Aquino , S. A. do Monte , F. Plasser , P. G. Szalay , H. Lischka , F. B. Machado , Phys. Chem. Chem. Phys. 2025, 27, 7916. 40165494 10.1039/d5cp00339c

[69] W. C. Chen , Y. C. Cheng , J. Phys Chem. A 2020, 124, 7644. 32864966 10.1021/acs.jpca.0c06482

[70] A. Czader , E. R. Bittner , J. Chem. Phys. 2008, 128, 035101. 18205523 10.1063/1.2821384

[71] R. Binder , S. Römer , J. Wahl , I. Burghardt , J. Chem. Phys. 2014, 141, 014101. 25005271 10.1063/1.4880415

