# Supplementary material for: Rationalising Exciton Interactions in Aggregates Based on the Transition Density
Source: Chemistry. 2025 Aug 14;31(52):e01570. doi: 10.1002/chem.202501570 (PMC12444747; doi:10.1002/chem.202501570)
Supplement: Supplementary file 1 — Supporting Information [file CHEM-31-e01570-s001.pdf]

Contents

S1: Charge transfer couplings **2**
Note S1.1: Evaluation of two-electron terms . . . 3
Note S1.2: Monomer orbitals and charge transfer integrals . . . 4
Note S1.3: Relation between the transition density coupling and CT coupling . . . 5

S2: Anthracene dimer **6**

S3: PDI dimer **9**

List of Tables

S1 Vertical excitation energies (E_x, eV) and oscillator strengths (f) for the stacked anthracene dimer at a distance of 4 Å for different shifts along the y-axis (short axis of the molecule) . . . 6
S2 Vertical excitation energies (E_x, eV) and oscillator strengths (f) for the stacked anthracene dimer at a distance of 4 Å for different shifts along the x-axis (long axis of the molecule) . . . 7
S3 S_1 vertical excitation energies in eV of the PDI dimer at different shifts along the x- and y-axis for an intermolecular distance of 3.3 Å. . . . 9
S4 S_2 vertical excitation energies in eV of the PDI dimer at different shifts along the x- and y-axis for an intermolecular distance of 3.3 Å. . . . 10
S5 Oscillator strengths ($f(S_1)$) of the S_1-state of the PDI dimer at different shifts along the x- and y-axis for an intermolecular distance of 3.3 Å. . . . 11

List of Figures

S1 Origin of charge-transfer contributions to the exciton splitting in a homodimer considering the case where the dark ^1A_g state becomes stabilised, thus, forming an H-aggregate: (a) splitting of the monomer frontier orbitals forming the dimer orbitals; (b) orbital transitions leading to the bright and dark excitons. . . . 2
S2 Analysis of the stacked anthracene dimer (d_z = 4.0 Å) with displacement along the short molecular axis: (a) vertical excitation energies (E_x) of the lowest dark and bright excited states; (b) charge transfer character of these states; (c) energy decomposition of the difference in excitation energy (ΔE_x) into one-electron ($\Delta h'$), two-electron (ΔE_2) and Coulomb (ΔJ_2) terms. . . . 8
S3 Analysis of the stacked PDI dimer (d_z = 3.3 Å) with displacement along the long molecular axis: (a) vertical excitation energies (E_x) of the lowest dark and bright excited states; (b) charge transfer character of these states; (c) energy decomposition of the difference in excitation energy (ΔE_x) into one-electron ($\Delta h'$), two-electron (ΔE_2) and Coulomb (ΔJ_2) terms. . . . 12

S1: Charge transfer couplings

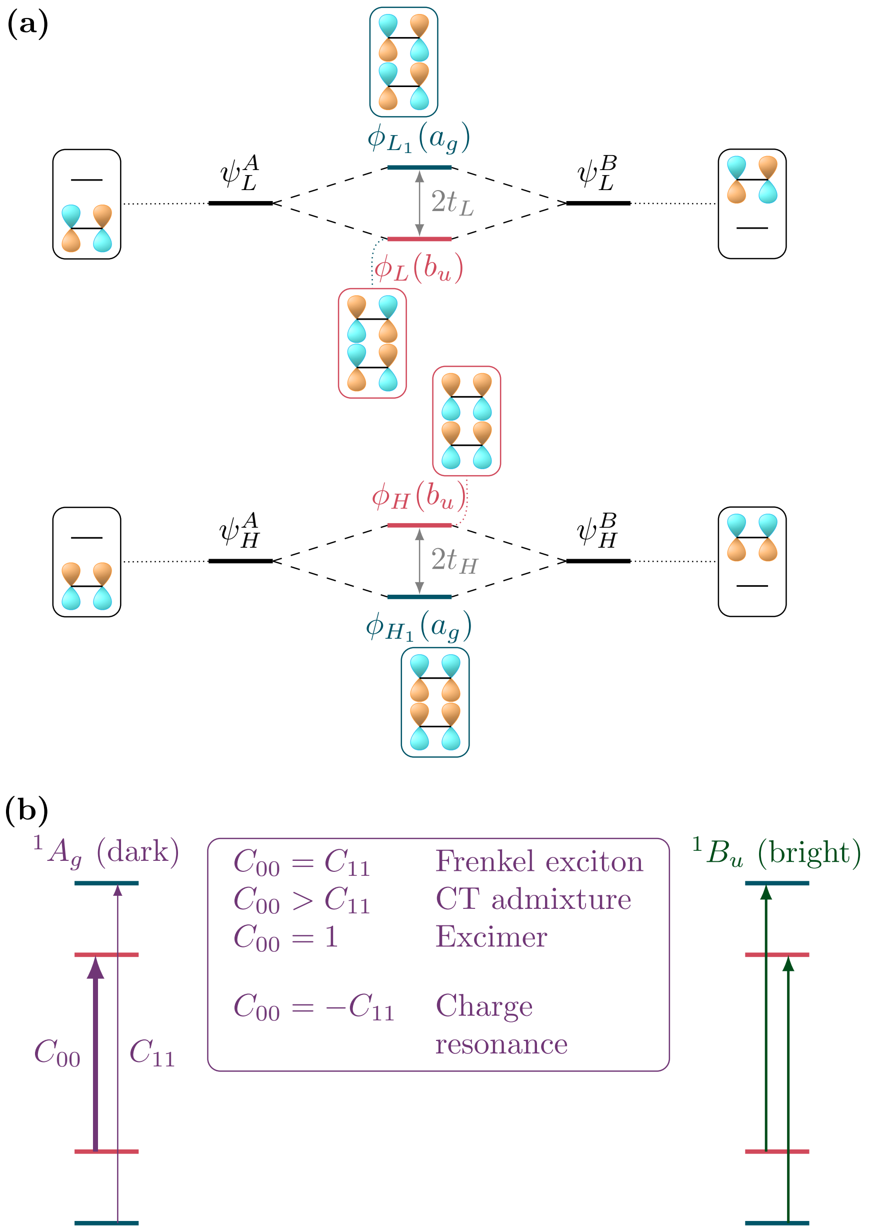

Figure S1: Origin of charge-transfer contributions to the exciton splitting in a homodimer considering the case where the dark ^1A_g state becomes stabilised, thus, forming an H-aggregate: (a) splitting of the monomer frontier orbitals forming the dimer orbitals; (b) orbital transitions leading to the bright and dark excitons.

Note S1.1: Evaluation of two-electron terms

It is the purpose of this section to derive an expression for the two-electron energy terms associated to CT mixing. Ignoring spin, for simplicity, the 1TDM in the monomer basis is given as

$$\gamma_t(r_h, r_e) = \frac{1}{2}\left(C_{00} + C_{11}\right)\left[\psi_H^A(r_h)\psi_L^A(r_e) + \psi_H^B(r_h)\psi_L^B(r_e)\right] + \frac{1}{2}\left(C_{00} - C_{11}\right)\left[-\psi_H^A(r_h)\psi_L^B(r_e) + \psi_H^B(r_h)\psi_L^A(r_e)\right] \tag{S1}$$

We are first interested in the K_2 term. The above 1TDM expression has to be inserted into

$$K_2 = -c_{HF}\iint \frac{\gamma_t(r_h, r_e)^2}{r_{he}}\mathrm{d}r_h\mathrm{d}r_e \tag{S2}$$

Initially this leads to a sum of 16 individual electron repulsion integrals (ERIs) of the general form

$$\left(\psi_H^C\psi_H^D \mid \psi_L^E\psi_L^F\right) = \iint \frac{\psi_H^C(r_h)\psi_H^D(r_h)\psi_L^E(r_e)\psi_L^F(r_e)}{r_{he}}\mathrm{d}r_h\mathrm{d}r_e \tag{S3}$$

To simplify the result, we neglect all differential overlap contributions meaning that we only include terms where $C = D$ and $E = F$. Reviewing Eq. (S1), this means that only the four terms where each orbital pair is multiplied with itself remains. Thus, we are left with the expression

$$\begin{aligned} K_2 = -\frac{c_{HF}}{4}\left(C_{00} + C_{11}\right)^2 &\left[(\psi_H^A\psi_H^A \mid \psi_L^A\psi_L^A) + (\psi_H^B\psi_H^B \mid \psi_L^B\psi_L^B)\right] \\ &- \frac{c_{HF}}{4}\left(C_{00} - C_{11}\right)^2\left[(\psi_H^A\psi_H^A \mid \psi_L^B\psi_L^B) + (\psi_H^B\psi_H^B \mid \psi_L^A\psi_L^A)\right] = \\ &- \frac{c_{HF}}{2}\left(C_{00} + C_{11}\right)^2 J_{AA} - \frac{c_{HF}}{2}\left(C_{00} - C_{11}\right)^2 J_{AB} \end{aligned} \tag{S4}$$

Here J_{AA} is the HOMO/LUMO Coulomb integral on one monomer and J_{AB} is the intermolecular Coulomb integral. First, considering the pure Frenkel exciton state ($C_{00} = C_{11} = 1/\sqrt{2}$), one obtains

$$\widetilde{K}_2 = -c_{HF}J_{AA} \tag{S5}$$

Thus, for the energy shift of the CT-mixed state with respect to the pure Frenkel state, one obtains

$$\Delta K_2 = K_2 - \widetilde{K}_2 = -\frac{c_{HF}}{2}\left(C_{00} - C_{11}\right)^2\left(J_{AB} - J_{AA}\right) \tag{S6}$$

Proceeding next to the J_2 term, we can first write the transition density as

$$\rho_t(r) = \gamma_t(r, r) = \frac{1}{2}\left(C_{00} + C_{11}\right)\left[\psi_H^A(r)\psi_L^A(r) + \psi_H^B(r)\psi_L^B(r)\right] + \frac{1}{2}\left(C_{00} - C_{11}\right)\left[-\psi_H^A(r)\psi_L^B(r) + \psi_H^B(r)\psi_L^A(r)\right] \tag{S7}$$

If we, again, neglect the differential overlap contributions, then only the first half of this remains and the transition density becomes

$$\rho_t(r) = \frac{1}{2}\left(C_{00} + C_{11}\right)\left[\psi_H^A(r)\psi_L^A(r) + \psi_H^B(r)\psi_L^B(r)\right] \tag{S8}$$

Inserting this expression into the definition of J_2

$$J_2 = \iint \frac{\rho_t(r_1)\rho_t(r_2)}{r_{12}}\mathrm{d}r_1\mathrm{d}r_2 \tag{S9}$$

leads to the four ERIs

$$\begin{aligned} J_2 = \frac{1}{4}\left(C_{00} + C_{11}\right)^2 &\left[(\psi_H^A\psi_L^A \mid \psi_H^A\psi_L^A) + (\psi_H^B\psi_L^B \mid \psi_H^B\psi_L^B)\right] + \\ &\frac{1}{4}\left(C_{00} + C_{11}\right)^2\left[(\psi_H^A\psi_L^A \mid \psi_H^B\psi_L^B) + (\psi_H^B\psi_L^B \mid \psi_H^A\psi_L^A)\right] = \\ &\frac{1}{2}\left(C_{00} + C_{11}\right)^2\left[K_{AA} + (\psi_H^A\psi_L^A \mid \psi_H^B\psi_L^B)\right] \end{aligned} \tag{S10}$$

using the symbol K_{AA} to denote the HOMO/LUMO exchange integral on the monomer. The reference value for the pure locally excited (LE) Frenkel exciton state ($C_{00} = C_{11} = 1/\sqrt{2}$) is (ignoring spin)

$$\widetilde{J}_2 = K_{AA} + (\psi_H^A \psi_L^A \mid \psi_H^B \psi_L^B) \tag{S11}$$

The energy shift of the CT-coupled state with respect to the LE state is

$$\Delta J_2 = J_2 - \widetilde{J}_2 = \frac{1}{2}\left(C_{00} - C_{11}\right)^2 \left[-K_{AA} - (\psi_H^A \psi_L^A \mid \psi_H^B \psi_L^B)\right] \tag{S12}$$

In summary, the overall shift for the two-electron terms is

$$\Delta E_2 = \Delta K_2 + \Delta J_2 = \frac{1}{2}\left(C_{00} - C_{11}\right)^2 \left[-c_{HF}(J_{AB} - J_{AA}) - K_{AA} - (\psi_H^A \psi_L^A \mid \psi_H^B \psi_L^B)\right] \tag{S13}$$

Note that in this expression the XC_2 term was ignored (but it could be evaluated analogously to the J_2 term). To simplify Eq. (S13), we first note that the energies of an LE and CT state are given as

$$E_{\mathrm{LE}} = \epsilon_L^A - \epsilon_H^A - c_{HF} J_{AA} + K_{AA} \tag{S14}$$

$$E_{\mathrm{CT}} = \epsilon_L^B - \epsilon_H^A - c_{HF} J_{AB} \tag{S15}$$

The difference between these two expressions (assuming that $\epsilon_L^B = \epsilon_L^A$) is

$$E_{\mathrm{CT}} - E_{\mathrm{LE}} = -c_{HF}(J_{AB} - J_{AA}) - K_{AA}. \tag{S16}$$

We also note that the Coulomb coupling for the pure excitonic states is given as

$$2\widetilde{J}_2^{AB} = (\psi_H^A \psi_L^A \mid \psi_H^B \psi_L^B) \tag{S17}$$

Combining Eqs (S13), (S16), and (S17), now gives the final equation for the two-electron terms

$$\Delta E_2 = \frac{1}{2}\left(C_{00} - C_{11}\right)^2 \left[E_{\mathrm{CT}} - E_{\mathrm{LE}} - 2\widetilde{J}_2^{AB})\right] \tag{S18}$$

Note S1.2: Monomer orbitals and charge transfer integrals

Within this section, we discuss the symmetry relations between the monomer MOs and their relation to the sign of the HOMO and LUMO charge transfer integrals. As a starting point, we need to set the symmetry of the monomer MOs. Below, we will start with the following phase relations, as also shown in Figs 2 and S1.

$$\psi_H^B = \hat{C}_i \psi_H^A \tag{S19}$$

$$\psi_L^B = \hat{C}_i \psi_L^A \tag{S20}$$

In other words, the orbitals on monomer B are generated by starting with the orbitals from A and inverting them through the inversion center of the dimer. Using these monomer MOs, we can now form the *gerade* and *ungerade* linear combinations to form the dimer MOs

$$\phi_M^g = 2^{-1/2}(\psi_M^A + \psi_H^B) = 2^{-1/2}(1 + \hat{C}_i)\psi_M^A \tag{S21}$$

$$\phi_M^u = 2^{-1/2}(\psi_M^A - \psi_H^B) = 2^{-1/2}(1 - \hat{C}_i)\psi_M^A \tag{S22}$$

where $M = H, L$. To show that these orbitals are indeed *gerade* and *ungerade*, respectively, we can write

$$\hat{C}_i \phi_M^g = 2^{-1/2}(\hat{C}_i + 1)\psi_M^A = +\phi_M^g \tag{S23}$$

$$\hat{C}_i \phi_M^u = 2^{-1/2}(\hat{C}_i - 1)\psi_M^A = -\phi_M^u \tag{S24}$$

At this point we do not know which one of ϕ_H^g and ϕ_H^u is the HOMO and which one the HOMO–1 (and similarly for the LUMO and LUMO+1). To determine the ordering, we note that these orbitals are created by diagonalisation of a matrix of the form

$$\begin{pmatrix} \epsilon_M^A & t_M \\ t_M & \epsilon_M^A \end{pmatrix} \tag{S25}$$

producing the following energies for the delocalised orbitals

$$\epsilon_H^g = \epsilon_H^A + t_H \qquad \epsilon_L^g = \epsilon_L^A + t_L \tag{S26}$$

$$\epsilon_H^u = \epsilon_H^A - t_H \qquad \epsilon_L^u = \epsilon_L^A - t_L. \tag{S27}$$

One has a J-aggregate if the HOMO/LUMO transition is bright, and this is the case if the HOMO is of u symmetry and the LUMO of g symmetry or *vice versa*. Thus, a J-aggregate is obtained if either

$$\epsilon_H^g < \epsilon_H^u \wedge \epsilon_L^g < \epsilon_L^u \Leftrightarrow t_H < 0 \wedge t_L < 0 \tag{S28}$$

(where $\wedge$ is the logical "and" sign) or

$$\epsilon_H^g > \epsilon_H^u \wedge \epsilon_L^g > \epsilon_L^u \Leftrightarrow t_H > 0 \wedge t_L > 0. \tag{S29}$$

The other two cases correspond to an H-aggregate. In summary this means that

$$\text{J-type coupling: } t_H \times t_L > 0 \tag{S30}$$

$$\text{H-type coupling: } t_H \times t_L < 0 \tag{S31}$$

and these are the same relations derived by Spano and co-workers using a perturbation theory approach starting from monomer states [8].

Note S1.3: Relation between the transition density coupling and CT coupling

In a final step, we want to explain why one can generally assume that CT coupling follows the same rules as derived for the transition densities. To do so, we first write the explicit expression for the charge transfer integrals

$$t_M = \langle \psi_M^A | \hat{f} | \psi_M^B \rangle \approx -f \langle \psi_M^A | \psi_M^B \rangle \tag{S32}$$

where $\hat{f}$ is the Fock operator; in the second part of this expression we take the common assumption that transfer integrals are roughly proportional to orbital overlap and that in-phase overlap stabilises the MO energies.

In a next step, we assume that the overall overlap density, $\psi_H^A(r)\psi_H^B(r)$, is either predominantly positive or predominantly negative everywhere in space (as illustrated in Figs 2 and S1). From Eq. (S32) it follows that the charge transfer integral has the same sign as the overlap density and we can differentiate the following four cases

$$\psi_H^A(r)\psi_H^B(r) > 0 \wedge \psi_L^A(r)\psi_L^B(r) > 0 \;\Rightarrow\; t_H < 0 \wedge t_L < 0 \;\Leftrightarrow\; \text{J-type coupling} \tag{S33}$$

$$\psi_H^A(r)\psi_H^B(r) < 0 \wedge \psi_L^A(r)\psi_L^B(r) > 0 \;\Rightarrow\; t_H > 0 \wedge t_L < 0 \;\Leftrightarrow\; \text{H-type coupling} \tag{S34}$$

$$\psi_H^A(r)\psi_H^B(r) > 0 \wedge \psi_L^A(r)\psi_L^B(r) < 0 \;\Rightarrow\; t_H < 0 \wedge t_L > 0 \;\Leftrightarrow\; \text{H-type coupling} \tag{S35}$$

$$\psi_H^A(r)\psi_H^B(r) > 0 \wedge \psi_L^A(r)\psi_L^B(r) < 0 \;\Rightarrow\; t_H > 0 \wedge t_L > 0 \;\Leftrightarrow\; \text{J-type coupling} \tag{S36}$$

where the type of coupling was determined according to Eqs (S30) and (S31). These four equations can be summarised as

$$\psi_H^A(r)\psi_H^B(r)\psi_L^A(r)\psi_L^B(r) > 0 \;\Rightarrow\; \text{J-type coupling} \tag{S37}$$

$$\psi_H^A(r)\psi_H^B(r)\psi_L^A(r)\psi_L^B(r) < 0 \;\Rightarrow\; \text{H-type coupling} \tag{S38}$$

After inserting inserting $\rho_t^A(r) = \psi_H^A(r)\psi_L^A(r)$ and $\rho_t^B(r) = \psi_H^B(r)\psi_L^B(r)$, we obtain

$$\rho_t^A(r)\rho_t^B(r) > 0 \;\Rightarrow\; \text{J-type coupling} \tag{S39}$$

$$\rho_t^A(r)\rho_t^B(r) < 0 \;\Rightarrow\; \text{H-type coupling} \tag{S40}$$

which can be rewritten as

$$\rho_t^A(r)\hat{C}_i\rho_t^A(r) > 0 \;\Rightarrow\; \text{J-type coupling} \tag{S41}$$

$$\rho_t^A(r)\hat{C}_i\rho_t^A(r) < 0 \;\Rightarrow\; \text{H-type coupling} \tag{S42}$$

These expressions now follow the rules derived for the Coulomb coupling. In particular this can be seen from Fig. 9. To start, the transition density on B is generated *via* inversion starting from the transition density on A. J-type coupling is now obtained if the overlap is positive (if red lobes are near read lobes); H-type coupling if the overlap is negative (if blue lobes are near red lobes).

S2: Anthracene dimer

Table S1: Vertical excitation energies (E_x, eV) and oscillator strengths (f) for the stacked anthracene dimer at a distance of 4 Å for different shifts along the y-axis (short axis of the molecule)

d_y/Å	$E_x(S_1)$	$f(S_1)$	$E_x(S_2)$	$f(S_2)$	$E_x(T_1)$	$E_x(T_2)$
0.0	3.579	0.0	4.005	0.122	2.379	2.501
0.5	3.609	0.0	3.995	0.124	2.409	2.499
1.0	3.697	0.0	3.969	0.129	2.445	2.493
1.5	3.807	0.0	3.934	0.137	2.471	2.484
2.0	3.902	0.0	3.907	0.152	2.480	2.490
2.5	3.894	0.176	3.949	0.0	2.476	2.497
3.0	3.900	0.203	3.967	0.0	2.478	2.499
3.5	3.917	0.229	3.978	0.0	2.482	2.502
4.0	3.937	0.247	3.987	0.0	2.490	2.504
4.5	3.951	0.259	3.992	0.0	2.493	2.506

Table S2: Vertical excitation energies (E_x, eV) and oscillator strengths (f) for the stacked anthracene dimer at a distance of 4 Å for different shifts along the x-axis (long axis of the molecule)

d_y/Å	$E_x(S_1)$	$f(S_1)$	$E_x(S_2)$	$f(S_2)$	$E_x(T_1)$	$E_x(T_2)$
0.0	3.579	0.000	4.005	0.122	2.379	2.501
0.5	3.644	0.000	4.024	0.173	2.426	2.499
1.0	3.809	0.000	3.989	0.126	2.480	2.493
1.1	3.845	0.000	3.978	0.123	2.488	2.492
1.2	3.878	0.000	3.968	0.121	2.491	2.494
1.3	3.903	0.000	3.958	0.120	2.490	2.498
1.3	3.910	0.000	3.954	0.120	2.489	2.498
1.4	3.913	0.000	3.951	0.120	2.489	2.498
1.4	3.911	0.000	3.947	0.121	2.489	2.498
1.5	3.905	0.000	3.944	0.121	2.489	2.497
1.5	3.896	0.000	3.942	0.121	2.489	2.496
1.6	3.885	0.000	3.940	0.122	2.489	2.494
1.6	3.873	0.000	3.938	0.123	2.489	2.492
1.7	3.861	0.000	3.938	0.123	2.490	2.490
1.8	3.837	0.000	3.937	0.126	2.484	2.490
2.0	3.795	0.000	3.944	0.132	2.472	2.493
2.3	3.759	0.000	3.968	0.150	2.458	2.497
2.4	3.755	0.000	3.978	0.160	2.456	2.498
2.5	3.755	0.000	3.988	0.172	2.455	2.498
2.6	3.760	0.000	3.996	0.183	2.455	2.498
2.7	3.767	0.000	4.001	0.192	2.457	2.498
3.0	3.808	0.000	3.989	0.182	2.467	2.495
3.5	3.900	0.000	3.939	0.161	2.485	2.489
3.6	3.915	0.000	3.930	0.160	2.483	2.492
3.8	3.920	0.160	3.937	0.000	2.481	2.497
4.0	3.916	0.163	3.946	0.000	2.481	2.500
4.2	3.919	0.168	3.946	0.000	2.484	2.500
4.4	3.929	0.175	3.942	0.000	2.488	2.499
4.5	3.937	0.180	3.941	0.000	2.491	2.499
4.6	3.939	0.000	3.944	0.184	2.494	2.499
4.8	3.939	0.000	3.960	0.194	2.498	2.499
5.0	3.941	0.000	3.974	0.204	2.499	2.502
5.2	3.945	0.000	3.982	0.211	2.499	2.504
5.4	3.950	0.000	3.984	0.214	2.500	2.504
5.6	3.955	0.000	3.981	0.214	2.500	2.503
6.0	3.962	0.000	3.970	0.213	2.498	2.502
6.5	3.964	0.215	3.968	0.000	2.496	2.502
7.0	3.971	0.220	3.972	0.001	2.499	2.503

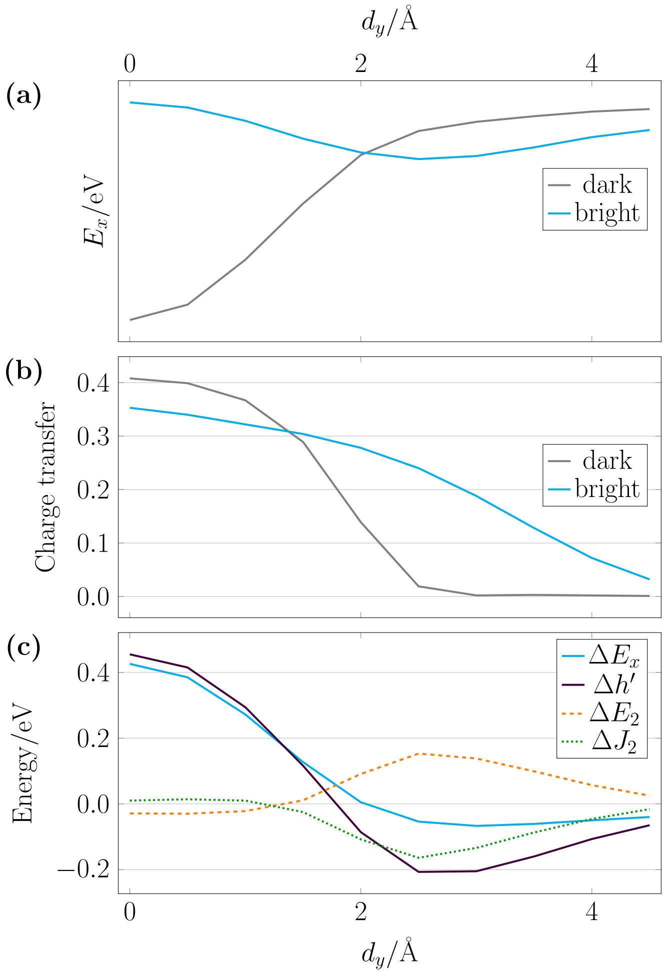

Figure S2: Analysis of the stacked anthracene dimer ($d_z = 4.0$ Å) with displacement along the short molecular axis: (a) vertical excitation energies (E_x) of the lowest dark and bright excited states; (b) charge transfer character of these states; (c) energy decomposition of the difference in excitation energy (ΔE_x) into one-electron ($\Delta h'$), two-electron (ΔE_2) and Coulomb (ΔJ_2) terms.

S3: PDI dimer

Table S3: S_1 vertical excitation energies in eV of the PDI dimer at different shifts along the x- and y-axis for an intermolecular distance of 3.3 Å.

d_y/Å<br>d_x/Å	0.0	0.5	1.0	1.5	2.0	2.5	3.0	3.5
0.0	1.833	1.980	2.346	2.704	2.543	2.462	2.554	2.703
0.5	1.949	2.086	2.425	2.736	2.561	2.515	2.624	2.770
1.0	2.240	2.352	2.629	2.699	2.572	2.614	2.769	2.845
1.5	2.572	2.658	2.703	2.560	2.546	2.684	2.840	2.709
2.0	2.493	2.566	2.535	2.463	2.526	2.726	2.750	2.579
2.5	2.456	2.531	2.520	2.468	2.549	2.760	2.737	2.568
3.0	2.523	2.595	2.658	2.575	2.616	2.787	2.829	2.685
3.5	2.635	2.700	2.853	2.732	2.699	2.793	2.913	2.843
4.0	2.429	2.525	2.751	2.871	2.758	2.770	2.867	2.930
4.5	2.312	2.417	2.667	2.843	2.799	2.761	2.826	2.907
5.0	2.377	2.474	2.709	2.832	2.838	2.812	2.870	2.915
5.5	2.579	2.659	2.849	2.856	2.869	2.894	2.962	2.935
6.0	2.817	2.875	2.966	2.895	2.894	2.962	3.022	2.967
6.5	2.853	2.905	2.972	2.923	2.907	2.958	3.015	2.978
7.0	2.772	2.826	2.944	2.940	2.922	2.942	2.989	2.977
7.5	2.756	2.807	2.923	2.979	2.942	2.944	2.977	2.995
8.0	2.791	2.836	2.934	2.979	2.953	2.953	2.977	2.994
8.5	2.853	2.887	2.953	2.971	2.952	2.958	2.981	2.992
9.0	2.918	2.936	2.963	2.959	2.951	2.965	2.983	2.988
9.5	2.954	2.959	2.961	2.955	2.957	2.972	2.983	2.984
10.0	2.966	2.965	2.962	2.960	2.967	2.979	2.984	2.983
10.5	2.971	2.971	2.970	2.970	2.977	2.985	2.988	2.988
11.0	2.979	2.978	2.979	2.981	2.985	2.990	2.993	2.995
11.5	2.986	2.986	2.986	2.988	2.992	2.996	2.999	3.002
12.0	2.992	2.992	2.993	2.995	2.998	3.001	3.004	3.007

Table S4: S_2 vertical excitation energies in eV of the PDI dimer at different shifts along the x- and y-axis for an intermolecular distance of 3.3 Å.

d_y/Å d_x/Å	0.0	0.5	1.0	1.5	2.0	2.5	3.0	3.5
0.0	2.811	2.807	2.762	2.751	2.711	2.800	2.856	2.808
0.5	2.812	2.821	2.793	2.785	2.739	2.829	2.895	2.848
1.0	2.756	2.796	2.839	2.797	2.787	2.865	2.956	2.927
1.5	2.622	2.685	2.813	2.830	2.799	2.848	2.948	2.977
2.0	2.737	2.668	2.737	2.825	2.778	2.798	2.890	2.971
2.5	2.698	2.636	2.714	2.840	2.787	2.793	2.877	2.969
3.0	2.859	2.792	2.769	2.894	2.839	2.846	2.930	3.018
3.5	2.670	2.744	2.871	2.927	2.867	2.893	2.988	3.067
4.0	2.741	2.799	2.920	2.894	2.848	2.902	3.020	3.012
4.5	2.838	2.888	2.922	2.922	2.826	2.912	3.022	2.938
5.0	2.934	2.956	2.897	2.949	2.848	2.946	3.009	2.940
5.5	3.003	2.991	2.917	2.987	2.892	2.976	3.023	3.005
6.0	2.960	3.006	2.997	2.952	2.915	2.978	3.030	3.010
6.5	2.998	3.016	2.994	2.936	2.936	3.007	3.027	2.994
7.0	3.032	3.011	2.965	2.967	2.971	3.036	3.030	3.001
7.5	3.074	3.059	3.021	2.997	3.017	3.060	3.053	3.015
8.0	3.096	3.097	3.082	3.057	3.057	3.077	3.077	3.058
8.5	3.078	3.088	3.099	3.088	3.079	3.085	3.087	3.079
9.0	3.063	3.076	3.096	3.097	3.089	3.090	3.091	3.087
9.5	3.075	3.085	3.101	3.103	3.097	3.095	3.095	3.092
10.0	3.097	3.102	3.109	3.108	3.103	3.101	3.100	3.096
10.5	3.111	3.113	3.114	3.112	3.108	3.105	3.103	3.099
11.0	3.115	3.115	3.115	3.112	3.110	3.107	3.104	3.100
11.5	3.115	3.114	3.114	3.111	3.109	3.107	3.104	3.101
12.0	3.112	3.112	3.111	3.110	3.108	3.106	3.103	3.100

Table S5: Oscillator strengths ($f(S_1)$) of the S_1-state of the PDI dimer at different shifts along the x- and y-axis for an intermolecular distance of 3.3 Å.

d_y/Å d_x/Å	0.0	0.5	1.0	1.5	2.0	2.5	3.0	3.5
0.0	0.000	0.000	0.000	0.285	0.000	0.000	0.000	0.000
0.5	0.000	0.000	0.000	0.293	0.000	0.000	0.000	0.000
1.0	0.000	0.000	0.000	0.000	0.000	0.000	0.000	0.000
1.5	0.000	0.000	0.000	0.000	0.000	0.000	0.000	0.000
2.0	0.495	0.441	0.000	0.000	0.000	0.000	0.000	0.000
2.5	0.634	0.582	0.000	0.000	0.000	0.000	0.000	0.000
3.0	0.783	0.727	0.000	0.000	0.000	0.000	0.000	0.000
3.5	0.875	0.806	0.502	0.000	0.000	0.000	0.000	0.000
4.0	0.000	0.000	0.000	0.000	0.000	0.000	0.000	0.000
4.5	0.000	0.000	0.000	0.682	0.000	0.000	0.000	0.000
5.0	0.000	0.000	0.000	0.959	0.980	0.000	0.000	1.275
5.5	0.000	0.000	0.000	1.193	1.191	0.000	0.000	1.503
6.0	0.000	0.000	1.529	1.389	1.381	0.000	0.001	1.728
6.5	1.267	1.369	0.000	0.003	1.560	1.717	2.094	0.000
7.0	1.445	1.491	1.819	0.000	1.755	1.822	2.045	0.000
7.5	1.649	1.680	1.893	2.190	1.951	1.952	2.073	2.163
8.0	1.820	1.854	2.038	2.232	2.079	2.067	2.142	2.194
8.5	1.972	2.019	2.183	2.239	2.142	2.149	2.206	2.227
9.0	2.151	2.196	2.274	2.241	2.192	2.215	2.248	2.240
9.5	2.285	2.296	2.292	2.255	2.239	2.259	2.264	2.242
10.0	2.311	2.308	2.294	2.275	2.271	2.277	2.268	2.247
10.5	2.310	2.308	2.299	2.289	2.284	2.280	2.268	2.252
11.0	2.308	2.306	2.301	2.294	2.286	2.277	2.266	2.254
11.5	2.301	2.300	2.297	2.290	2.281	2.271	2.261	2.250
12.0	2.290	2.289	2.286	2.280	2.272	2.263	2.254	2.243

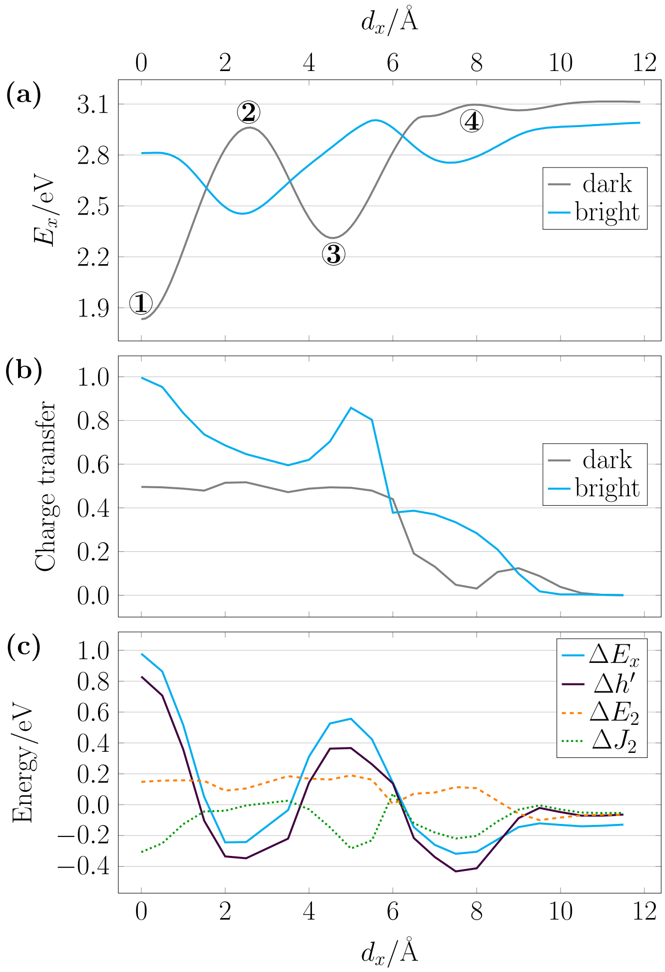

Figure S3: Analysis of the stacked PDI dimer ($d_z = 3.3$ Å) with displacement along the long molecular axis: (a) vertical excitation energies (E_x) of the lowest dark and bright excited states; (b) charge transfer character of these states; (c) energy decomposition of the difference in excitation energy (ΔE_x) into one-electron ($\Delta h'$), two-electron (ΔE_2) and Coulomb (ΔJ_2) terms.

References

[8] H. Yamagata, C. M. Pochas, F. C. Spano, *J. Phys. Chem. B* **2012**, *116*, 14494.
